# Analysis of dim-light responses in rod and cone photoreceptors with altered calcium kinetics

**DOI:** 10.1007/s00285-023-02005-4

**Published:** 2023-10-12

**Authors:** Annia Abtout, Jürgen Reingruber

**Affiliations:** 1 Institute of Biology, Ecole Normale Supérieure, Paris, France; 2 INSERM, U1024, Paris, France

**Keywords:** Signal transduction, Photoreceptor, Rod, Cone, Calcium dynamics, Mathematical modelling, Asymptotic analysis, 37N25, 92C37, 92C40

## Abstract

Rod and cone photoreceptors in the retina of vertebrates are the primary sensory neurons underlying vision. They convert light into an electrical current using a signal transduction pathway that depends on Ca$^{2+}$ feedback. It is known that manipulating the Ca$^{2+}$ kinetics affects the response shape and the photoreceptor sensitivity, but a precise quantification of these effects remains unclear. We have approached this task in mouse retina by combining numerical simulations with mathematical analysis. We consider a parsimonious phototransduction model that incorporates negative Ca$^{2+}$ feedback onto the synthesis of cyclic GMP, and fast buffering reactions to alter the Ca$^{2+}$ kinetics. We derive analytic results for the photoreceptor functioning in sufficiently dim light conditions depending on the photoreceptor type. We exploit these results to obtain conceptual and quantitative insight into how response waveform and amplitude depend on the underlying biophysical processes and the Ca$^{2+}$ feedback. With a low amount of buffering, the Ca$^{2+}$ concentration changes in proportion to the current, and responses to flashes of light are monophasic. With more buffering, the change in the Ca$^{2+}$ concentration becomes delayed with respect to the current, which gives rise to a damped oscillation and a biphasic waveform. This shows that biphasic responses are not necessarily a manifestation of slow buffering reactions. We obtain analytic approximations for the peak flash amplitude as a function of the light intensity, which shows how the photoreceptor sensitivity depends on the biophysical parameters. Finally, we study how changing the extracellular Ca$^{2+}$ concentration affects the response.

## Introduction

Vision in most vertebrates starts with the absorption of light by rod and cone photoreceptors located in the retina of the eye (Ebrey and Koutalos 2001). Rods are highly sensitive to light and sustain vision under scotopic light conditions, whereas cones are much less sensitive compared to rods, but they adapt and maintain vision even under brightest illumination (Burns and Baylor 2001). The perception of light starts with the absorption of light by photopigments located the outer segment (OS) of rod and cone photoreceptors, which initiates a phototransduction cascade that leads to an electrical current response (for reviews see (Burns and Pugh 2010; Arshavsky and Burns 2012; Arshavsky et al. 2002; Pugh and Lamb 2000)). Many of the biochemical processes that participate in this signal transduction pathway are modulated by Ca$^{2+}$ feedback (Vinberg et al. 2018; Koch and Dell’Orco 2013; Nakatani et al. 2002; Korenbrot and Rebrik 2002; Rispoli 1998). Ca$^{2+}$ feedback not only enables photoreceptors to adapt their sensitivity to increasing light intensities in order to avoid early saturation (Yau and Nakatani 1985; Nakatani and Yau 1988; Matthews et al. 1988; Nakatani and Yau 1988; Matthews 1991; Koutalos and Yau 1996; Fain et al. 2001; Pugh et al. 1999), but this feedback also affects the light response in darkness (Klaus et al. 2021; Burns et al. 2002; Sakurai et al. 2011; Koutalos et al. 1995; Lagnado et al. 1992; Torre et al. 1986). For example, in most species, dark-adapted rods show monophasic responses to brief flashes of light (Hamer et al. 2003; Field and Rieke 2002; Pugh and Lamb 2000, 1993). However, when the Ca$^{2+}$ kinetics are distorted and slowed down by the application of exogenous buffers, biphasic flash responses have been observed in amphibian rods (Torre et al. 1986; Lamb et al. 1986; Torre et al. 1986; Korenbrot and Miller 1989; Rieke and Baylor 1998), in primate and guinea pig rods (Field and Rieke 2002; Burns et al. 2002; Matthews 1991), and in mouse rods (Burns et al. 2002; Makino et al. 2004). For cones, biphasic responses have even been observed without the application of exogenous buffers (Korenbrot 2012; Holcman and Korenbrot 2005; Schneeweis and Schnapf 1999; Schnapf et al. 1990; Baylor et al. 1987; Schnapf et al. 1987; Matthews et al. 1990; Nunn et al. 1984). Recently it has been claimed that biphasic cone responses are not physiological but generated by experimental conditions that distorted the Ca$^{2+}$ dynamics (Cao et al. 2014).

The dynamics of the free Ca$^{2+}$ concentration in the OS depends on influx through cyclic GMP (cGMP) gated cyclic nucleotide-gated (CNG) channels, efflux via NCKX exchangers, and buffering reactions (Pugh and Lamb 2000). The impact of buffering has been modelled either using explicit equations for the interactions of free Ca$^{2+}$ with a combination of slow and fast buffers (Forti et al. 1989; Tamura et al. 1991; Hamer et al. 2003; Dell’Orco et al. 2009; Chen et al. 2010; Invergo et al. 2014; Astakhova et al. 2015), or by considering a simplified approach with an effective buffering capacity $B_{ca}$ (Sneyd and Tranchina 1989; Tranchina et al. 1991; Nikonov et al. 1998; Pugh and Lamb 2000; Caruso et al. 2005; Gross et al. 2012a, b; Reingruber et al. 2013; Lamb and Kraft 2016, 2020) (in Korenbrot (2012) a Ca$^{2+}$-dependent value for $B_{ca}$ has been assumed). Although the latter models are only valid for fast buffering kinetics, it turns our that they are nevertheless often sufficient to reproduce experimental data.

Since Ca$^{2+}$ feedback is ubiquitous in biology, the Ca$^{2+}$ kinetics and the effect of buffering have been studied both analytically and numerically for many biological systems, see for example (Falcke 2004; Wagner and Keizer 1994; Sneyd et al. 1995). In phototransduction, fitting procedures and numerical simulations have been mostly applied to adjust the Ca$^{2+}$ kinetics to experimental data and to investigate the impact of buffering (Forti et al. 1989; Tamura et al. 1991; Hamer and Tyler 1995; Nikonov et al. 1998; Moriondo and Rispoli 2003; Hamer et al. 2003; Korenbrot 2012)(in addition to numerical simulations, some first-order analysis has been performed in (Schnapf et al. 1990; Nikonov et al. 1998)). Although simulations show that changing the Ca$^{2+}$ kinetics affects the response dynamics and the photoreceptor sensitivity, without analytic results it remains difficult to obtain a precise conceptual and quantitative understanding.

In this work, we combined numerical simulations with mathematical analysis to systematically study how the Ca$^{2+}$ kinetics affects waveform and amplitude of light responses in mouse rod and cone photoreceptors. We started from a parsimonious phototransduction model that comprises the principal transduction processes that are known to be relevant for dark-adapted photoreceptors, and that we have previously calibrated with experimental data from mouse rods and cones (Reingruber et al. 2020; Abtout et al. 2021). Whereas in previous work we assumed that under physiological conditions the free Ca$^{2+}$ concentration changes in proportion to the current, we now consider the general case where the free Ca$^{2+}$ concentration depends on influx via CNG channels, efflux via exchangers, and buffering. To keep the analysis comprehensible, we on fast buffering reactions and we ignore the impact of slow buffering. This is in line with models that use a buffering capacity $B_{ca}$. To obtain analytic results, we performed a linear-response analysis for light intensities that are sufficiently dim such that changes of the cGMP and Ca$^{2+}$ concentrations, and subsequently of the current, are small compared to their corresponding steady state values in darkness. Because cones are much less sensitive to light than rods (Ingram et al. 2016), this analysis is valid up to much brighter light intensities for cones compared to rods. Thus, the definition of dim light depends on the photoreceptor sensitivity and therefore differs between rods and cones. For cones, dim light comprises a range of light intensities that is much larger than the one for rods. We combined analytic results and numerical simulations to investigate the light response as a function of the Ca$^{2+}$ kinetics. With a low amount of buffering, we find that the Ca$^{2+}$ concentration changes in proportion to the current, and we recover our previous results from (Reingruber et al. 2020; Abtout et al. 2021). In the low buffering range the waveforms of brief flashes of light are monophasic. As the amount of buffering increases, the Ca$^{2+}$ dynamics becomes delayed with respect to the dynamics of cGMP and current, and biphasic waveforms emerge that contain a damped oscillation. A phase space analysis shows that the transition from monophasic to biphasic responses depends on the ratio between the rate $\mu _{ca}$ that controls the the Ca$^{2+}$ kinetics, and the dark turnover rate of cGMP $\beta _d$. Because $\beta _d$ changes among species, and between rods and cones (Reingruber et al. 2020; Pugh and Lamb 2000), the same Ca$^{2+}$ kinetics does not entail similar waveforms in rods and cones. We use our analytic results to dissect the contributions of the various biophysical processes to the waveform, and to identify the processes that limit the response recovery. We further derive an analytic approximation for the peak amplitude as a function of the flash intensity, which reveals how the photoreceptor sensitivity depends on the underlying biophysical parameters and the Ca$^{2+}$ kinetics. Finally, we investigate the response of cones to stimulations with longer steps of light, and we study the effect of changing the extracellular Ca$^{2+}$ concentration.

## Materials and methods

### Phototransduction model

We start from the phototransduction model from (Abtout et al. 2021; Reingruber et al. 2020), and we consider here a more general Ca$^{2+}$ dynamics where the free Ca$^{2+}$ concentration does not necessarily change in proportion to the circulating current. In short, light activates the visual pigment $R^*$ with a rate that is proportional to the light intensity $\phi (t)$ times the collecting area $\kappa$. $R^*$ activates the G-protein transducin $T^*$ with a rate $k_{act}$, and deactivates with rate $\mu _{rh}$. $T^*$ activates phosphodiesterase (PDE) $P^*$ with a rate $k_{tr}$, and deactivates with rate $\mu _{tr}$. Because the deactivation of $T^*$ is linked to the activation of PDE, we have $k_{tr}=\mu _{tr}$ (to keep the analysis most general, we distinguish between $k_{tr}$ and $\mu _{tr}$). $P^*$ deactivates with rate $\mu _{pde}$, and hydrolyses the cytosolic second messenger cyclic GMP (cGMP) with rate constant $\beta _{sub}$. cGMP gates the opening of CNG channels, in the OS membrane. The rate by which the cGMP concentration $c_{cg}$ is synthesized by guanylate cyclase (GC) depends on the free Ca$^{2+}$ concentration $c_{ca}$, $\alpha (c_{ca})=\alpha _{max} \frac{r_\alpha c_{ca}^{n_\alpha } + K_{\alpha }^{n_\alpha }}{c_{ca}^{n_\alpha } + K_{\alpha }^{n_\alpha }}$, where $r_\alpha =\frac{\alpha _{min}}{\alpha _{max}}$. The GC activity is modulated via an intermediate step where Ca$^{2+}$ binds to GCAPs proteins (Mendez et al. 2001; Burns et al. 2002). Ca$^{2+}$ feedback not only modulates the cyclase activity, it also affects the deactivation rate of an activated photopigment via binding to recoverin, which inhibits the phosphorylation of the photopigment by rhodopsin kinase (Klenchin et al. 1995; Chen et al. 1995, 2012). However, in this work we focus on the Ca$^{2+}$ feedback to the cyclase, which is the most effective feedback under dim-light conditions (Burns et al. 2002; Sakurai et al. 2011; Koutalos et al. 1995). We assume that the Ca$^{2+}$-dependent modulation of photopigment deactivation occurs on a much slower time scale, which affects adaptation process but can be neglected in first approximation for flash responses.

The Ca$^{2+}$ content of the OS changes due to influx via CNG channels and efflux via electrogenic NCKX exchangers. We adopt the convention that inward currents are negative. The CNG current is $I_{ch} = I_{ch,max}p_{ch}$, where $p_{ch}(c_{cg}) =\frac{c_{cg}^{n_{ch}}}{c_{cg}^{n_{ch}}+ K_{ch}^{n_{ch}}}$ is the cGMP dependent fraction of open channels, and $K_{ch}$ is the cGMP concentration where 50% of the channels are open. The CNG current carried by Ca$^{2+}$ is $I_{ch,ca} = f_{ch,ca} I_{ch}$, where $f_{ch,ca}$ is the fraction of the current carried by Ca$^{2+}$. The influx of Ca$^{2+}$ via the CNG channels is $\frac{I_{ch,ca}}{2e^+}$ (inward fluxes are negative). The exchanger current as a function of the free Ca$^{2+}$ concentration is $I_{ex} = I_{ex,sat} p_{ex}$, where $p_{ex}(c_{ca})= \frac{c_{ca}^{n_{ex}} }{c_{ca}^{n_{ex}} + K_{ex}^{n_{ex}}}$ is the exchanger saturation level, and $I_{ex,sat}$ is the saturating current at high $c_{ca}$. The exchanger stoichiometry is such that the extrusion of a single Ca$^{2+}$ ion leads to the influx of a positive charge $e^+$. Thus, the exchanger current is negative, and the Ca$^{2+}$ efflux is $-\frac{I_{ex}}{e^+}$. Although the exchanger cooperativity $n_{ex}$ is found to be one (Pugh and Lamb 2000), we keep a general $n_{ex}$ for the analysis to see how this parameter affects the results. But for numerical evaluations we use $n_{ex}=1$ (Table 1). Ca$^{2+}$ regulates the activity of many proteins in the outer segment (Fain et al. 2001; Nakatani et al. 2002; Korenbrot and Rebrik 2002; Rispoli 1998), which contributes to Ca$^{2+}$ buffering. With $N_b$ buffer species $b_i$ ($i=1,\ldots N_{b}$), the Ca$^{2+}$ concentrations that are bound to these buffers are $c_{ca,b_i}$. With standard buffer kinetics (Keener and Sneyd 1998; Wagner and Keizer 1994), the equations for the total and buffered Ca$^{2+}$ concentrations are

1 $$\begin{aligned} \begin{array}{rcl} \displaystyle F_A V_{os} \frac{d}{dt} \left( c_{ca} + \sum _{i=1}^{N_b}c_{ca,b_i}\right) &{}=&{} \displaystyle - \left( \frac{I_{ch,ca}}{2} - I_{ex}\right) \\ \displaystyle \frac{d}{dt} c_{ca,b_i} &{}=&{} \displaystyle \mu _{b_i}\left( \frac{(c_{b_i,tot} -c_{ca,b_i}) c_{ca} }{K_{b_i}}- c_{ca,b_i}\right) ,\, \, i=1,\ldots N_{b}. \end{array} \end{aligned}$$

$c_{b_i,tot}$ are total buffer concentrations, $K_{b_i}$ are dissociation constants, $\mu _{b_i}$ are dissociation rates, $F_A= 9.65\times 10^{-5}\frac{s pA}{\mu M \mu m^3}$ is the Faraday constant, and $V_{os}$ is the outer segment volume. We assume that the buffer kinetics are fast (the times $\mu _{b_i}^{-1}$ are small compared to the time scale for the change in the free Ca$^{2+}$ concentration due to the current), in which case the amount of buffered Ca$^{2+}$ becomes a function of the free Ca$^{2+}$ concentration, $c_{ca,b_i} =\frac{c_{b_i,tot}c_{ca}}{c_{ca}+K_{b_i}}$ (quasi steady-state approximation) (Keener and Sneyd 1998; Wagner and Keizer 1994). Equation 1 now simplifies to

2 $$\begin{aligned} \displaystyle F_A V_{os} \left( 1 + \sum _{i=1}^{N_b} \frac{c_{b_i,tot} K_{b_i}}{(c_{ca} +K_{b_i})^2}\right) \frac{d}{dt} c_{ca} = \displaystyle -\left( \frac{I_{ch,ca}}{2} - I_{ex} \right) . \end{aligned}$$

The closed system of transduction equations is

3 $$\begin{aligned} \begin{array}{rcl} \displaystyle \frac{d}{dt} R^*&{}=&{} \displaystyle \phi (t) \kappa - \mu _{rh} R^* \\ \displaystyle \frac{d}{dt} T^* &{}=&{} \displaystyle k_{act} R^* - \mu _{tr}T^* \\ \displaystyle \frac{d}{dt} P^* &{}=&{} \displaystyle k_{tr} T^* -\mu _{pde} P^* \\ \displaystyle \frac{d}{dt} c_{cg} &{}=&{} \displaystyle \alpha _{max} \frac{r_\alpha c_{ca}^{n_\alpha } + K_{\alpha }^{n_\alpha }}{c_{ca}^{n_\alpha } + K_{\alpha }^{n_\alpha }} - (\beta _d +\beta _{sub} P^*) c_{cg} \\ \displaystyle \frac{d}{dt} c_{ca} &{}=&{} \displaystyle -\frac{1}{F_A V_{os}} \left( 1 + \sum _{i=1}^{N_b} \frac{c_{b_i,tot} K_{b_i}}{(c_{ca} +K_{b_i})^2}\right) ^{-1} \left( \frac{I_{ch,ca}(c_{cg})}{2} - I_{ex}(c_{ca}) \right) , \end{array} \end{aligned}$$

where $\beta _d$ is the basal cGMP hydrolysis rate in darkness. The total current is

4 $$\begin{aligned} I= I_{ch}(c_{cg})+I_{ex}(c_{ca}) = I_{ch,max}\frac{c_{cg}^{n_{ch}}}{c_{cg}^{n_{ch}}+ K_{ch}^{n_{ch}}} + I_{ex,sat} \frac{c_{ca}^{n_{ex}} }{c_{ca}^{n_{ex}} + K_{ex}^{n_{ex}}}. \end{aligned}$$

To study the dynamics with respect to the steady state in darkness, we use steady state values $c_{ca,0}$, $c_{cg,0}$ and $I_0$ to introduce normalized variables and parameters (Sneyd and Tranchina 1989). Steady state values can either be computed with Eq. 3, or experimental values can be used. We replace the parameters $\alpha _{max}$, $I_{ch,max}$ and $I_{ex,sat}$ (which are not well known) with the steady state quantities $c_{ca,0}$, $c_{cg,0}$ and $I_0$. For example, we have $I_{ex,sat} = I_{ex}(c_{ca,0}) \frac{c_{ca,0}^{n_{ex}} + K_{ex}^{n_{ex}}}{c_{ca,0}^{n_{ex}}}$ and $I_{ch,max} = I_{ch}(c_{cg,0}) \frac{c_{cg,0}^{n_{ch}}+ K_{ch}^{n_{ch,0}}}{c_{cg,0}^{n_{ch}}}$. From the steady state conditions in darkness we obtain $\alpha (c_{ca,0}) = \beta _d c_{cg,0}$ and $I_{ch,ca}(c_{cg,0}) = f_{ch,ca} I_{ch}(c_{cg,0}) = 2 I_{ex}(c_{ca,0})$. The dark current is $I_0= I_{ch}(c_{cg,0})+I_{ex}(c_{ca,0})= \frac{f_{ch,ca}+2}{2} I_{ch}(c_{cg,0}) = \frac{f_{ch,ca}+ 2}{f_{ch,ca}} I_{ex}(c_{ca,0})$. We label normalized variables and parameters with a hat, and we define ${\hat{c}}_{cg}=\frac{c_{cg}}{c_{cg,0}}$, ${\hat{c}}_{ca}=\frac{c_{ca}}{c_{ca,0}}$, ${\hat{c}}_{b_i,tot}=\frac{c_{bi,tot}}{c_{ca,0}}$, ${\hat{K}}_{ch}=\frac{K_{ch}}{c_{cg,0}}$, ${\hat{K}}_{ex}=\frac{K_{ex}}{c_{ca,0}}$, ${\hat{K}}_{\alpha }=\frac{K_{\alpha }}{c_{ca,0}}$, ${\hat{K}}_{b_i}=\frac{K_{b_i}}{c_{ca,0}}$. We further introduce the dark buffering capacities $B_{b_i}= \frac{c_{b_i,tot} K_{b_i}}{(c_{ca,0}+ K_{b_i})^2}$, and the overall capacity $B_{ca} = \sum _{i=1}^{N_b} B_{b_i}$. With the new variables ${\tilde{P}}^*= \beta _{sub}P^*$, ${\tilde{T}}^*= \frac{\beta _{sub}k_{tr}}{\mu _{pde}} T^*$, ${\tilde{R}}^*= \frac{ \beta _{sub} k_{act} k_{tr}}{\mu _{pde} \mu _{tr}} R^*$, we obtain from Eq. 3

5 $$\begin{aligned} \begin{array}{rcl} \displaystyle \frac{d}{dt} {\tilde{R}}^*&{}=&{} \displaystyle \mu _{rh} ( \phi (t) \kappa \xi - {\tilde{R}}^* ) \\ \displaystyle \frac{d}{dt} {\tilde{T}}^* &{}=&{} \displaystyle \mu _{tr} ( {\tilde{R}}^* - {\tilde{T}}^* ) \\ \displaystyle \frac{d}{dt} {\tilde{P}}^* &{}=&{} \displaystyle \mu _{pde} ( {\tilde{T}}^* -{\tilde{P}}^* ) \\ \displaystyle \frac{d}{dt} {\hat{c}}_{cg} &{}=&{} \displaystyle \beta _{d} {\hat{\alpha }} ({\hat{c}}_{ca}) - (\beta _d +{\tilde{P}}^*) {\hat{c}}_{cg} \\ \displaystyle \frac{d}{dt} {\hat{c}}_{ca} &{}=&{} \displaystyle \frac{1+B_{ca}}{1 + \sum _{i=1}^{N_b} B_{b_i} \frac{(1+{\hat{K}}_{b_i})^2}{({\hat{c}}_{ca} +{\hat{K}}_{b_i})^2}} \mu _{ca}\left( {\hat{p}}_{ch}({\hat{c}}_{cg}) -{\hat{p}}_{ex}({\hat{c}}_{ca})\right) , \end{array} \end{aligned}$$

with the gain $\xi = \frac{k_{act}\beta _{sub} k_{tr} }{ \mu _{rh}\mu _{pde} \mu _{tr}}$ and

6 $$\begin{aligned} \begin{array}{rcl} \displaystyle \mu _{ca} &{}=&{} \displaystyle -\frac{1}{(1+B_{ca})F_AV_{os}} \frac{I_{ex}(c_{ca,0})}{c_{ca,0}} = -\frac{1}{(1+B_{ca})F_AV_{os}} \frac{f_{ch,ca}}{f_{ch,ca}+2} \frac{I_0}{c_{ca,0}} \\ \displaystyle {\hat{\alpha }} ({\hat{c}}_{ca}) &{}=&{} \displaystyle \frac{\alpha (c_{ca})}{\alpha (c_{ca,0})}=\frac{1 + {\hat{K}}_{\alpha }^{n_\alpha }}{ r_\alpha + {\hat{K}}_{\alpha }^{n_\alpha }} \frac{r_\alpha {\hat{c}}_{ca}^{n_\alpha } + {\hat{K}}_{\alpha }^{n_\alpha }}{{\hat{c}}_{ca}^{n_\alpha } + {\hat{K}}_{\alpha }^{n_\alpha }}\\ \displaystyle {\hat{p}}_{ch} ({\hat{c}}_{cg})&{}=&{} \displaystyle \frac{p_{ch}(c_{cg})}{p_{ch}(c_{cg,0})} = \frac{1 + {\hat{K}}_{ch}^{n_{ch}} }{{\hat{c}}_{cg}^{n_{ch}} + {\hat{K}}_{ch}^{n_{ch}}} {\hat{c}}_{cg}^{n_{ch}}\\ \displaystyle {\hat{p}}_{ex} ({\hat{c}}_{ca}) &{}=&{} \displaystyle \frac{p_{ex}(c_{ca})}{p_{ex}(c_{ca,0})} = \frac{1+{\hat{K}}_{ex}^{n_{ex}}}{{\hat{c}}_{ca}^{n_{ex}}+{\hat{K}}_{ex}^{n_{ex}}} {\hat{c}}_{ca}^{n_{ex}} . \end{array} \end{aligned}$$

The initial conditions in darkness with $\phi =0$ are $\tilde{R}^*={\tilde{T}}^*={\tilde{P}}^*=0$ and ${\hat{c}}_{cg}= {\hat{c}}_{ca} = \hat{\alpha }= {\hat{p}}_{ch} = {\hat{p}}_{ex} =1$. The current normalised by the dark current is

7 $$\begin{aligned} \begin{array}{rcl} \displaystyle {\hat{I}} &{}=&{} \displaystyle {\hat{I}}_{ch}+ {\hat{I}}_{ex} = \frac{I_{ch}(c_{cg,0})}{I_0} \frac{I_{ch}(c_{cg})}{I_{ch}(c_{cg,0})} + \frac{I_{ex}(c_{ca,0})}{I_0} \frac{I_{ex }(c_{ca})}{I_{ex}(c_{ca,0})} \\ &{}=&{} \displaystyle \frac{2}{f_{ch,ca}+2} {\hat{p}}_{ch}+ \frac{f_{ch,ca}}{f_{ch,ca}+2} {\hat{p}}_{ex} . \end{array} \end{aligned}$$

We further introduce the normalised current ${\hat{i}} = \displaystyle 1- {\hat{I}}$, which is zero in darkness. With Eq. 7 we get

8 $$\begin{aligned} \displaystyle {\hat{i}} = {\hat{i}}_{ch} + {\hat{i}}_{ex} = \frac{2}{f_{ch,ca}+2} \left( 1- {\hat{p}}_{ch}\right) +\frac{f_{ch,ca}}{f_{ch,ca} + 2} \left( 1- {\hat{p}}_{ex}\right) . \end{aligned}$$

${\hat{i}}$ is usually used in the literature for the light response. Although ${\hat{i}}$ is referred to as a current, one has to keep in mind that ${\hat{i}}$ is the current change with respect to the dark current. ${\hat{I}}$ and ${\hat{i}}$ have complementary properties, for example, whereas ${\hat{I}}$ decreases after the light is switched on, ${\hat{i}}$ increases.

Before proceeding with the analysis, we add some remarks:

1. Because we study the light response in darkness, we defined the buffering capacities using the steady state Ca$^{2+}$ concentration in darkness. In presence of a background light, we would use the steady state Ca$^{2+}$ concentration corresponding to this background light.
2. By definition, the expression $(1+B_{ca}) /\left( {1 + \sum _{i=1}^{N_b} B_{b_i} \frac{(1+{\hat{K}}_{b_i})^2}{({\hat{c}}_{ca} +{\hat{K}}_{b_i})^2}}\right)$ is one in darkness, and can be neglected in first order. Hence, for low activation we have $\frac{d}{dt} {\hat{c}}_{ca} \approx \mu _{ca}\left( {\hat{p}}_{ch} -{\hat{p}}_{ex}\right)$.
3. Due to the normalisation, Eq. 5 depends only on kinetic parameters that govern the dynamics. Equation 5 therefore is convenient to study the response dynamics as a function of the light intensity since it contains less parameters than Eq. 3. For example, Eq. 5 shows that the activation rates $k_{act}$, $k_{tr}$ and $\beta _{sub}$ affect the light response only as the product $k_{act} k_{tr} \beta _{sub}$.
4. Eq. 5 has been obtained assuming that the normalisation values $c_{ca,0}$ and $c_{cg,0}$ are the steady state concentrations in darkness. In Eq. 5 the steady state in darkness with ${\tilde{P}}^*=0$ is therefore always ${\hat{c}}_{cg}= {\hat{c}}_{ca}=1$. To modify these values, we have to add additional parameters to Eq. 5. For example, to investigate how changing the cyclase activity alters the steady state, we introduce $\zeta = \frac{\alpha (c_{ca,0})}{\beta _d c_{cg,0}}$, such that $\frac{d}{dt} {\hat{c}}_{cg} = \beta _{d} \zeta {\hat{\alpha }} ({\hat{c}}_{ca}) - (\beta _d +{\tilde{P}}^*) {\hat{c}}_{cg}$. For $\zeta = 1$ we have ${\hat{c}}_{cg}= {\hat{c}}_{ca}=1$. For $\zeta \ne 1$, the steady state values are obtained by solving ${\hat{p}}_{ch}({\hat{c}}_{cg}) -{\hat{p}}_{ex}({\hat{c}}_{ca})=0$ with ${\hat{c}}_{cg}=\zeta {\hat{\alpha }}({\hat{c}}_{ca})$.

### Linear response analysis

The equations for PDE activation in Eq. 5 are linear and can be solved explicitly. The solution is

9 $$\begin{aligned} {\tilde{P}}^* = \kappa \xi \int _0^t \phi (s) G_p(t-s) ds, \end{aligned}$$

with the Green’s function

10 $$\begin{aligned} G_{pde}= \frac{\mu _{rh} \mu _{tr} \mu _{pde}}{(\mu _{rh} -\mu _{tr})(\mu _{rh}-\mu _{pde})} e^{-\mu _{rh}t} + (\mu _{rh} \leftrightarrow \mu _{tr}) + (\mu _{rh} \leftrightarrow \mu _{pde}).\quad \end{aligned}$$

The double-headed arrows in Eq. 10 signify that the second and third terms in the equation are identical to the first term with the exchange $\mu _{tr}$ and $\mu _{rh}$ in the second term, and $\mu _{pde}$ and $\mu _{rh}$ in the third term. The non-linear equations for ${\hat{c}}_{cg}$ and ${\hat{c}}_{ca}$ (Eq. 5) cannot be solved analytically for general $\phi (t)$. However, analytic results can be derived for dim light where the changes of ${\hat{c}}_{cg}$ and ${\hat{c}}_{ca}$ are small compared to the steady state values ${\hat{c}}_{cg}={\hat{c}}_{ca}=1$. For the analysis we introduce the new variables $y= -\ln {\hat{c}}_{cg}$ and $z=-\ln {\hat{c}}_{ca}$, which are both zero in darkness. With $y\ll 1$ and $z\ll 1$, by linearizing Eq. 5 we obtain the first-order system of equations

11 $$\begin{aligned} \begin{array}{rcl} \displaystyle \frac{d}{dt} \, y &{}=&{} \displaystyle {\tilde{P}}^*- \beta _{d} \left( y +{\hat{\alpha }}'_0 \nu \, u \right) \\ \displaystyle \frac{d}{dt}\, u &{}=&{} \displaystyle \beta _d r \left( y - u \right) , \end{array} \end{aligned}$$

where $$\nu u=z$$ and

12 $$\begin{aligned} \begin{array}{rcl} \displaystyle {\hat{\alpha }}'_0 &{}=&{} \displaystyle \frac{d}{dz}\hat{\alpha }(z)\Big |_{z=0} = -\frac{d}{d{\hat{c}}_{ca}}{\hat{\alpha }}({\hat{c}}_{ca})\Big |_{{\hat{c}}_{ca}=1} = \frac{ n_{\alpha } {\hat{K}}_{\alpha }^{n_\alpha }(1 - r_\alpha ) }{(1+{\hat{K}}_{\alpha }^{n_\alpha }) ({\hat{K}}_{\alpha }^{n_\alpha } +r_\alpha )} \\ \displaystyle \nu &{}=&{} \displaystyle \frac{n_{ch}}{n_{ex}} \frac{1+{\hat{K}}_{ex}^{n_{ex}}}{{\hat{K}}_{ex}^{n_{ex}}} \frac{ {\hat{K}}_{ch}^{n_{ch}} }{1+ {\hat{K}}_{ch}^{n_{ch}}} \\ \displaystyle r &{}=&{} \displaystyle \frac{\mu _{ca}}{\beta _d}\frac{n_{ex}{\hat{K}}_{ex}^{n_{ex}}}{1+{\hat{K}}_{ex}^{n_{ex}}} . \end{array} \end{aligned}$$

Note that ${\hat{\alpha }}'_0 \ge 0$. The solution of Eq. 11 with ${\tilde{P}}^*(t)$ from Eq. 9 is

13 $$\begin{aligned} \displaystyle y = \displaystyle \kappa \xi \sum _{i=1}^2 \zeta _{y,i} \int _0^t \phi (s) G_i(t-s) ds, \quad \displaystyle u = \displaystyle \kappa \xi \sum _{i=1}^2 \zeta _{u,i} \int _0^t \phi (s) G_i(t-s) ds ,\qquad \end{aligned}$$

where $\zeta _{y,1}=\frac{r-\lambda _1}{\lambda _2-\lambda _1}$, $\zeta _{y,2}=\frac{r-\lambda _2}{\lambda _1-\lambda _2}$, $\zeta _{u,1}=\frac{ r}{\lambda _2-\lambda _1}$, $\zeta _{u,2}=\frac{ r}{\lambda _1-\lambda _2}$. $\lambda _1 = \frac{1+r -\sqrt{(1-r)^2 -4r \nu {\hat{\alpha }}'_0 }}{2}$ and $\lambda _2 = \frac{1+r +\sqrt{(1-r)^2 -4r \nu {\hat{\alpha }}'_0 }}{2}$ are the eigenvalues of the matrix $$A=\begin{pmatrix} 1 &{} {\hat{\alpha }}'_0 \nu \\ - r &{} r \end{pmatrix}$$. The Green’s functions are ($\beta _i = \beta _d \lambda _i$, $i=1,2$)

14 $$\begin{aligned} \begin{array}{rcl} \displaystyle G_i &{}&{}= \displaystyle -\frac{ \mu _{rh} \mu _{tr} \mu _{pde} e^{-\mu _{rh}t}}{(\mu _{rh} -\mu _{tr})(\mu _{rh}-\mu _{pde})(\mu _{rh}-\beta _i )} \\ {} &{}&{} \qquad + (\mu _{rh} \leftrightarrow \mu _{tr}) + (\mu _{rh} \leftrightarrow \mu _{pde}) + (\mu _{rh} \leftrightarrow \beta _i ) \\ &{}&{}= \gamma _{rh,i} e^{-\mu _{rh}t} + \gamma _{tr,i} e^{-\mu _{tr}t} + \gamma _{pde,i} e^{-\mu _{pde}t} + \gamma _{\beta _i} e^{-\beta _i t} \end{array} \end{aligned}$$

(the double-headed arrows have the same significance as in Eq. 10).

Experimentally, photoreceptors are often studied by stimulating them with flashes or steps of light. To connect our analysis to such data, we compute the photoreceptor response to a light-step with duration $\Delta t$ and intensity $\phi$. The number of isomerisations produced by this stimulus is $R^*_0= \kappa \phi \Delta t$. From Eq. 13 we obtain $y=R^*_0 \xi g_y$, $u= R^*_0 \xi g_u$ and $z= R^*_0 \xi \nu g_u$, with

15 $$\begin{aligned} \displaystyle g_y = \displaystyle \sum _{i=1}^2 \zeta _{y,i}\frac{1}{\Delta t} \int _0^{\min (t,\Delta t)} G_i(t-s) ds, \quad \displaystyle g_u =\displaystyle \sum _{i=1}^2 \zeta _{u,i} \frac{1}{\Delta t} \int _0^{\min (t,\Delta t)} G_i(t-s) ds .\nonumber \\ \end{aligned}$$

From Eq. 8 we obtain in first order for the current

16 $$\begin{aligned} \begin{array}{rcl} \displaystyle {\hat{i}} &{}=&{} \displaystyle \frac{2}{f_{ch,ca}+2}\frac{ n_{ch} {\hat{K}}_{ch}^{n_{ch}} }{1+ {\hat{K}}_{ch}^{n_{ch}}} y + \frac{f_{ch,ca}}{f_{ch,ca}+2} \frac{n_{ex}{\hat{K}}_{ex}^{n_{ex}}}{1+{\hat{K}}_{ex}^{n_{ex}}} z \\ &{}=&{} \displaystyle R^*_0 \xi \frac{ n_{ch} {\hat{K}}_{ch}^{n_{ch}} }{1+ {\hat{K}}_{ch}^{n_{ch}}} \left( \frac{2}{f_{ch,ca}+2} g_y + \frac{f_{ch,ca}}{f_{ch,ca}+2} g_u\right) . \end{array} \end{aligned}$$

For $\Delta t\rightarrow 0$ we have $\frac{1}{\Delta t} \int _0^{\min (t,\Delta t)} G_i(t-s) \approx G_i(t)$. With Eq. 14 we obtain that ${\hat{i}}$ is a sum of exponentials,

17 $$\begin{aligned} {\hat{i}} = {\hat{i}}_{rh} + {\hat{i}}_{tr} +{\hat{i}}_{pde} + {\hat{i}}_{\beta } , \end{aligned}$$

with ${\hat{i}}_{rh}= R^*_0 c_{rh} e^{-\mu _{rh}t}$, ${\hat{i}}_{tr}=R^*_0 c_{tr} e^{-\mu _{tr}t}$, ${\hat{i}}_{pde}= R^*_0 c_{pde}e^{-\mu _{pde}t}$ and ${\hat{i}}_{\beta }= R^*_0 c_{\beta _1}e^{-\beta _1t} + R^*_0 c_{\beta _2} e^{-\beta _2t}$. The coefficients are

$$\begin{aligned} c_{rh}= \xi \frac{ n_{ch} {\hat{K}}_{ch}^{n_{ch}} }{1+ {\hat{K}}_{ch}^{n_{ch}}} \left( \frac{2}{f_{ch,ca}+2} \sum _{i=1}^2 \zeta _{y,i} \gamma _{rh,i} + \frac{f_{ch,ca}}{f_{ch,ca}+2} \sum _{i=1}^2 \zeta _{u,i} \gamma _{rh,i}\right) , \end{aligned}$$

and similar expressions apply for the other coefficients. For $R^*_0=1$ we obtain the single-photon response (SPR).

#### Steady state with constant light intensity

With constant $\phi$ we have ${\tilde{P}}^* = \xi \kappa \phi$. The steady state of Eq. 11 is $u=y$ and $y = \frac{{\tilde{P}}^*}{\beta _d(1+ \nu {\hat{\alpha }}'_0)} =\frac{\phi \kappa \xi }{\beta _d(1+ \nu {\hat{\alpha }}'_0)}$. With the steady state condition ${\hat{p}}_{ch} ={\hat{p}}_{ex}$ we obtain for the current in dim light

18 $$\begin{aligned} {\hat{i}} = 1- {\hat{p}}_{ch} \approx n_{ch} y = \frac{n_{ch} \kappa \xi \phi }{\beta _d(1+ \nu {\hat{\alpha }}'_0)}, \end{aligned}$$

where we used ${\hat{K}}_{ch} \gg 1$. For brighter light, the steady state is computed from Eq. 5 by numerically solving ${\hat{p}}_{ch}({\hat{c}}_{cg}) -{\hat{p}}_{ex}({\hat{c}}_{ca}) =0$ with ${\hat{c}}_{cg} = \frac{\beta _d {\hat{\alpha }} ({\hat{c}}_{ca})}{\beta _d +\tilde{P}^*}$.

#### Emergence of damped oscillations

We investigate how the current ${\hat{i}}_{\beta }$ in Eq. 17 changes as a function of the the Ca$^+$ kinetics. For this we very the rate $\mu _{ca}$, respectively the parameter $r\sim \frac{\mu _{ca}}{\beta _d}$. For $(1-r)^2 - 4r \nu {\hat{\alpha }}'_0 >0$ the eigenvalues $\lambda _1$ and $\lambda _2$ are real and positive. In this range the origin $y=z=0$ is a stable node and flash responses are monophasic (Strogatz 2018). For example, with fast Ca$^+$ kinetics (low amount of buffering such that $r \gg 1$) we find $\lambda _1 \approx 1 +\nu {\hat{\alpha }}'_0$, $\lambda _2 \approx r- \nu {\hat{\alpha }}'_0 \rightarrow \infty$ and ${\hat{i}}_{\beta } \sim e^{-\beta _d (1 +\nu {\hat{\alpha }}'_0) t}$. In the opposite limit of very slow Ca$^+$ kinetics (high amount of buffering) the free Ca$^{2+}$ concentration remains constant. For $r\rightarrow 0$ we have $\lambda _1 \approx (1+\nu {\hat{\alpha }}'_0 ) r\rightarrow 0$, $\lambda _2 \approx 1- \nu {\hat{\alpha }}'_0 r\rightarrow 1$, $\zeta _{y,1}\rightarrow 0$, $\zeta _{y,2}\rightarrow 1$, $\zeta _{u,1}\rightarrow 0$, $\zeta _{u,2}\rightarrow 0$, such that ${\hat{i}}_{\beta } \sim e^{-\beta _d t}$.

For $(1-r)^2 - 4r \nu {\hat{\alpha }}'_0 <0$ the eigenvalues $\lambda _1$ and $\lambda _2$ are complex conjugate with non-vanishing real part $\lambda _{re} =\frac{1+r}{2}$. In this range the origin is a stable spiral and ${\hat{i}}_{\beta }$ is a damped oscillation (biphasic response) (Strogatz 2018). Oscillations occur for $r_{1}< r <r_{2}$, where $r_1 = 1+2\nu {\hat{\alpha }}'_0 \left( 1- \sqrt{1+ \frac{1}{\nu {\hat{\alpha }}'_0 } } \right)$ and $r_2 = 1+2\nu {\hat{\alpha }}'_0 \left( 1+ \sqrt{1+ \frac{1}{\nu {\hat{\alpha }}'_0 }} \right)$ are the values where the origin changes between stable node and stable spiral. By writing $\lambda _{1} = \lambda _{re} - i \lambda _{im}$ and $\lambda _{2} = \lambda _{re} +i \lambda _{im}$, with $\lambda _{im} =\frac{ \sqrt{(r-r_1)(r_2-r)} }{2}$, we find

19 $$\begin{aligned} {\hat{i}}_{\beta } = R^*_0 c_{\beta _1}e^{-\beta _d \lambda _1t} + R^*_0 c_{\beta _2} e^{-\beta _d \lambda _2t}\displaystyle = R^*_0 a e^{-\beta _{damp}t} \cos (\omega t +\varphi ). \end{aligned}$$

The damping rate is $\beta _{damp}=\beta _d\lambda _{re} = \beta _d \frac{1+r}{2}$, and the oscillation rate is $\omega =\beta _d\lambda _{im} =\beta _d \frac{ \sqrt{(r-r_1)(r_2-r)} }{2}$. Because $c_{\beta _1}$ and $c_{\beta _2}$ are complex conjugate, the amplitude *a* and the phase $\varphi$ of the oscillation can be computed from $\frac{a}{2} e^{i\varphi }= c_{\beta _1} = \xi \frac{ n_{ch}{\hat{K}}_{ch}^{n_{ch}} }{1+ {\hat{K}}_{ch}^{n_{ch}}} \left( \frac{2}{f_{ch,ca}+2} \zeta _{y,1} \gamma _{\beta ,1} + \frac{f_{ch,ca}}{f_{ch,ca}+2} \zeta _{u,1} \gamma _{\beta ,1}\right)$.

Since $\lambda _1$ and $\lambda _2$ are hyperbolic with non-vanishing real parts, the Hartman-Grobmann theorem states that the analysis of the linearized system in Eq. 11 also faithfully reflects the behaviour of the non-linear system in Eq. 5 locally (Strogatz 2018; Ricardo 2020). For a steady background light $\phi$, Eq. 5 has a single fixed point (stable node or spiral), and no limit cycles and self-sustained oscillations exist. This ensures that the visual perception is driven by the light input. Given that Eq. 5 contains only negative feedback, the lack of limit cycles is not surprising (however, there are special conditions where a system with only negative feedback can exhibit Hopf-bifurcations (Reidl et al. 2006)).

### Changing the extracellular Ca$^{2+}$ concentration

We now investigate how the response changes when the extracellular Ca$^{2+}$ concentration is altered by a factor of $r_{ca,ex}$ with respect to the reference concentration that had been implicitly assumed for the previous computations. We assume that the CNG current carried by Ca$^{2+}$ changes in proportion to the extracellular Ca$^{2+}$ concentration. We checked that this is a valid assumption by using the more general Goldman-Hodgkin-Katz(GHK) equation (Keener and Sneyd 1998) to estimate the current change. We further assume that the CNG current not carried by Ca$^{2+}$ remains unaffected by changing extracellular Ca$^{2+}$. Hence, we have ${\hat{I}}_{ch,ca} = r_{ca,ex}f_{ch,ca}\frac{2}{f_{ch,ca}+2}{\hat{p}}_{ch}$ and ${\hat{I}}_{ch,{\overline{ca}}} = (1-f_{ch,ca}) \frac{2}{f_{ch,ca}+2} {\hat{p}}_{ch}$. The total CNG current is ${\hat{I}}_{ch}= {\hat{I}}_{ch,ca} + {\hat{I}}_{ch,{\overline{ca}}} = \frac{{\hat{I}}_{ch,ca}}{f'_{ch,ca}}$, where

20 $$\begin{aligned} f'_{ch,ca}= f_{ch,ca}\frac{r_{ca,ex}}{1+ \left( r_{ca,ex} -1\right) f_{ch,ca} } \end{aligned}$$

is the new fraction of the current that is carried by Ca$^{2+}$. The total CNG and exchanger current is

21 $$\begin{aligned} \begin{array}{rcl} \displaystyle {\hat{I}} = \displaystyle {\hat{I}}_{ch} + {\hat{I}}_{ex} = \frac{r_{ca,ex}f_{ch,ca}}{f'_{ch,ca}} \frac{2}{f_{ch,ca}+2}{\hat{p}}_{ch} + \frac{f_{ch,ca}}{f_{ch,ca}+2}{\hat{p}}_{ex} . \end{array} \end{aligned}$$

The equation for ${\hat{c}}_{ca}$ reads

22 $$\begin{aligned} \displaystyle \frac{d}{dt} {\hat{c}}_{ca} = \displaystyle \frac{1+B_{ca}}{1 + \sum _{i=1}^{N_b} B_{b_i} \frac{(1+{\hat{K}}_{b_i})^2}{({\hat{c}}_{ca} +{\hat{K}}_{b_i})^2}} \mu _{ca}\left( r_{ca,ex} {\hat{p}}_{ch} -{\hat{p}}_{ex}\right) . \end{aligned}$$

Thus, for $r_{ca,ex}\ne 1$ we have that ${\hat{c}}_{ca}={\hat{c}}_{cg}=1$ are not the steady state solutions in darkness. The new steady state values ${\hat{c}}_{ca,d}$ and ${\hat{c}}_{cg,d}={\hat{\alpha }} ({\hat{c}}_{ca,d})$ are obtained by solving the equation $r_{ca,ex} {\hat{p}}_{ch}({\hat{\alpha }} ({\hat{c}}_{ca,d})) -{\hat{p}}_{ex}({\hat{c}}_{ca,d}) =0$. With ${\hat{p}}_{ex} = r_{ca,ex} {\hat{p}}_{ch}$ we obtain from Eq. 21 for the new dark current

23 $$\begin{aligned} {\hat{I}}_d = \left( 1-\frac{3f_{ch,ca} (1-r_{ca,ex}) }{f_{ch,ca} +2} \right) \frac{1 + {\hat{K}}_{ch}^{n_{ch}} }{{\hat{c}}_{cg}^{n_{ch}} + {\hat{K}}_{ch}^{n_{ch}}} {\hat{c}}_{cg}^{n_{ch}}. \end{aligned}$$

We can use the new steady state values ${\hat{c}}_{ca,d} c_{ca,0}$, ${\hat{c}}_{cg,d} c_{cg,0}$ and ${\hat{I}}_d I_0$ to renormalize parameters. Together with the new value for $f_{ch,ca}$, we again obtain equations like Eqs. 5 and 6 to study the light response.

**Fig. 1:**
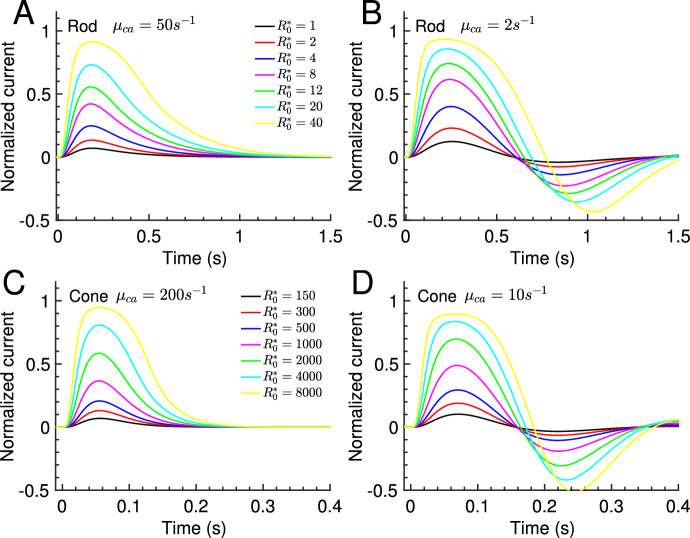
Simulations of flash responses for mouse rod and cone. The normalized current ${\hat{i}}$ from Eq. 8 is computed with Eq. 5 and parameters from Table 1 with a slow and a fast buffer species. **A** and **B** Family of flash responses with duration $\Delta t=5ms$ for a rod with fast and slow Ca$^{2+}$ kinetics $\mu _{ca}$, as indicated in the panels. The around 25 times higher buffering capacity $B_{ca}$ in **B** has been obtained by increasing both buffering concentrations by the same factor. The legend gives the number of isomerisations $R_0^*$ produced by the flash. **C**–**D** Similar to **A**–**B** but for a cone

## Results

Equation 5 shows that the Ca$^{2+}$ kinetics are determined by the effective rate $\mu _{ca}=\frac{1}{(1+B_{ca})F_AV_{os}} \frac{|I_{ex}(c_{ca,0}|)}{c_{ca,0}}$. Without buffering ($B_{ca} = 0$) and parameters from Table 1 we compute $\mu _{ca}\sim 1.6\times 10^3 s^{-1}$ for a rod, and $\mu _{ca}\sim 9.4\times 10^3\,s^{-1}$ for a cone. For large $\mu _{ca}$ we have the quasi steady state approximation ${\hat{p}}_{ch} \approx {\hat{p}}_{ex}$ and ${\hat{I}} \approx {\hat{p}}_{ch} \approx {\hat{p}}_{ex}$. Moreover, with ${\hat{K}}_{ex} \gg 1$ we have ${\hat{p}}_{ex}\approx {\hat{c}}_{ca}$ and ${\hat{c}}_{ca} \approx {\hat{I}}$. Thus, with fast kinetics we have that the Ca$^{2+}$ concentration changes in proportion to the current, and in this limit we recover the model and results from (Reingruber et al. 2020; Abtout et al. 2021).

With fast kinetics we observe only monophasic flash responses (Fig. 1A and C), whereas biphasic responses (damped oscillations) emerge as the Ca$^{2+}$ kinetics are slowed down (Fig. 1B and D). The simulations in Fig. 1 were performed with Eq. 5 and parameters from Table 1. We used one low and one high affinity buffer. The dissociation constant $K_{b_1}=3\mu M$ for the low affinity buffer corresponds to recoverin (Chen et al. 1995), whereas $K_{b_2}=0.14\mu M$ for the high affinity buffer corresponds to the GCAP proteins (Kawamura and Tachibanaki 2021). Since recoverin is the most abundant Ca$^{2+}$ buffer in the outer segment (Pugh and Lamb 2000; Kawamura and Tachibanaki 2021), it follows that under physiological conditions the buffering capacity of recoverin $B_{b_1}$ is much larger than the buffering capacity of the GCPAs $B_{b2}$. However, to show that our results are not biased towards low or high affinity buffers, we use $\frac{B_{b_1}}{B_{b_2}}=1$ for simulations such that low and high affinity buffers contribute equally to the total buffering capacity $B_{ca}= B_{b_1} +B_{b_2}$ (analytic results only depend on $B_{ca}$). Thus, for the simulations we altered the value of $\mu _{ca}$ by changing the buffering capacity $B_{ca}$ with the constraint that $\frac{B_{b_1}}{B_{ca}} = \frac{B_{b_2}}{B_{ca}}=0.5$. Physiologically this corresponds to changing both buffer concentrations by the same factor. For example, with parameters from Table 1 we obtain for a rod $\mu _{ca}=50s^{-1}$ using $B_{ca}=32$, and for a cone we get $\mu _{ca}=200s^{-1}$ with $B_{ca}=45$. We use $\mu _{ca}$ rather than $B_{ca}$ to characterize the Ca$^{2+}$ kinetics because $\mu _{ca}$ can be directly compared to the other rate constants of the model.

**Table 1:** Parameter values for the mouse rod and cone model

Parameter	Description	Rod	Cone
$\kappa$ $\left( \frac{\mu m^2}{photons}\right)$	Collecting area	0.28 (Abtout et al. 2021)	0.013 (Abtout et al. 2021)
$\xi = \frac{k_{act}\beta _{sub} k_{tr} }{ \mu _{rh}\mu _{pde} \mu _{tr}}$	Transduction gain of PDE activation	0.18 (Abtout et al. 2021)	0.0007 (Abtout et al. 2021)
$\beta _{d}$ ($s^{-1}$)	Dark turnover rate	4.1 (Reingruber et al. 2020)	11 (Reingruber et al. 2020)
$\mu _{rh}$ ($s^{-1}$)	Deactivation rate of activated photopigment	28 (Reingruber et al. 2020)	70 (Reingruber et al. 2020)
$\mu _{pde}$ ($s^{-1}$)	Deactivation rate of activated PDE	5 (Reingruber et al. 2020)	37.8 (Reingruber et al. 2020)
$\mu _{tr}$ ($s^{-1}$)	Deactivation rate of activated transducin	23.8 (Reingruber et al. 2020)	71 (Reingruber et al. 2020)
$K_{ex}$ ($\mu M$)	Sensitivity of NCKX exchanger to Ca$^{2+}$	1.6 (Pugh and Lamb 2000)	1.6
$K_{ch}$ ($\mu M$)	Sensitivity of CNG channels to cGMP	20 (Pugh and Lamb 2000)	20
$n_{ex}$	Exchanger cooperativity	1 (Reingruber et al. 2020)	1 (Reingruber et al. 2020)
$n_{ch}$	CNG channel cooperativity	2.5 (Reingruber et al. 2020)	2.5 (Reingruber et al. 2020)
$K_\alpha$ ($\mu M$)	Cyclase affinity to Ca$^{2+}$	0.26 (Reingruber et al. 2020)	0.26
$r_\alpha$	Ratio of minimal to maximal cyclase activity	0.033 (Peshenko and Dizhoor 2006; Peshenko 2011)	0.033
$n_\alpha$	Hill coefficient for the cyclase	2 (Reingruber et al. 2020)	2 (Reingruber et al. 2020)
$f_{ch,ca}$	Fraction of the CNG current carried by Ca$^{2+}$	0.12 (Gross et al. 2012a)	0.3 (Ohyama et al. 2000)
$I_0$ (*pA*)	Dark current	−15 (Reingruber et al. 2020)	−15 (Reingruber et al. 2020)
$c_{ca,d}$ ($\mu M$)	Dark Ca$^{2+}$ concentration	0.3 (Reingruber et al. 2020)	0.3 (Reingruber et al. 2020)
$c_{cg,d}$ ($\mu M$)	Dark cGMP concentration	4 (Reingruber et al. 2020)	4 (Reingruber et al. 2020)
$V_{os}$ ($\mu m^3$)	Outer segment volume	18 (Reingruber et al. 2013)	7.2 (Nikonov et al. 2006; Ingram et al. 2016)
$K_{b_1}$ ($\mu M$)	Dissociation constant of the low affinity Ca$^{2+}$ buffer	3 (Chen et al. 1995)	3
$K_{b_2}$ ($\mu M$)	Dissociation constant of the high affinity Ca$^{2+}$ buffer	0.14 (Kawamura and Tachibanaki 2021)	0.14

### Waveform and dynamics of flash responses

**Fig. 2:**
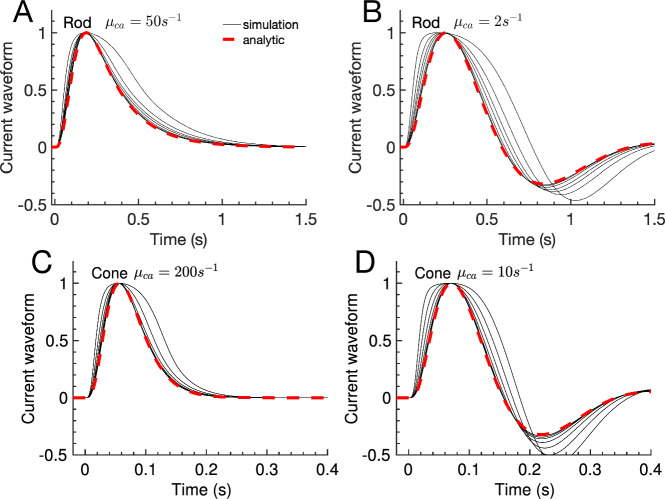
Flash waveform: simulation and analytic result. (**A**–**D**) The waveforms of the simulations in Fig. 1 are obtained by normalizing the responses to unit amplitude (black solid curves). The dashed red curves show the analytic waveform ${\hat{i}}/{\hat{i}}_{peak}$ computed with Eq. 16 (color figure online)

The current waveform is obtained by normalizing a flash response with its peak amplitude. The waveform therefore characterizes the current dynamics. The waveforms of the rod and cone simulations in Fig. 1 change only little up light intensities where the peak amplitude reaches values of the order 0.5 (Fig. 2, black solid lines). In this range, the analytic waveform $w = {{\hat{i}} }/{{\hat{i}}_{peak}}$ computed with Eq. 16 faithfully agrees with the simulations for rods and cones with fast and slow Ca$^{2+}$ dynamics (Fig. 2, black lines versus red dashed line).

**Fig. 3:**
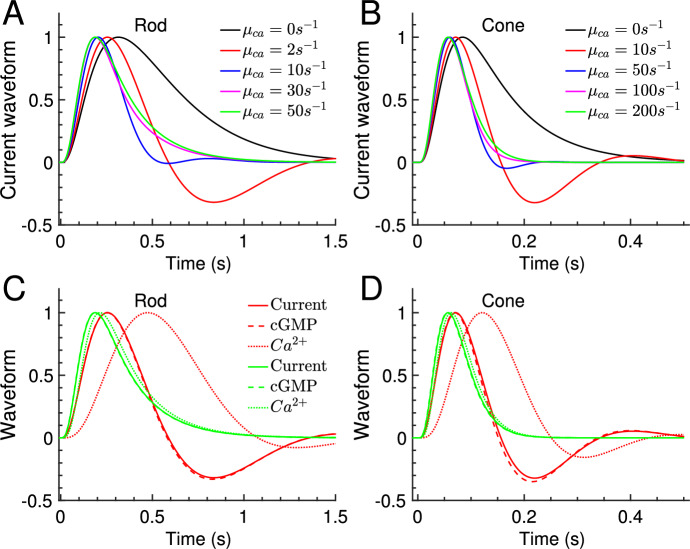
Flash waveforms for different Ca$^{2+}$ kinetics. **A** Comparison of the analytic current waveform ${\hat{i}}/{\hat{i}}_{peak}$ computed with Eq. 16 for a rod and for various values of $\mu _{ca}$ as indicated in the legend. **B** Similar to **A** but for a cone. **C** Comparison of the analytic rod waveforms for current (solid line), cGMP concentration (dashed lines) and Ca$^{2+}$ concentration (dotted lines) for $\mu _{ca}=50s^{-1}$ (green color) and $\mu _{ca}=2s^{-1}$ (red color). The current waveform is ${\hat{i}}/{\hat{i}}_{peak}$, the cGMP waveform is ${g_y}/{g_{y,peak}}$, and the Ca$^{2+}$ waveform is ${g_u}/{g_{u,peak}}$ (see Eq. 16). **D** Similar to (C) but for a cone with $\mu _{ca}=200s^{-1}$ (green color) and $\mu _{ca}=10s^{-1}$ (red color) (color figure online)

Next we used the analytic waveform to study how the the response changes as a function of $\mu _{ca}$. We further introduced waveforms for cGMP and Ca$^{2+}$ concentration, $w_{cgmp} = \frac{g_y }{g_{y,peak}}$ and $w_{ca} = \frac{g_u }{g_{u,peak}}$, respectively. Because the exchanger current is small, the overall waveform is determined by the cGMP dynamics (Fig. 3C-D, dashed versus solid curves). We find that waveforms are monophasic with fast Ca$^{2+}$ kinetics (Fig. 3A-B, green curves). In this limit the Ca$^{2+}$ concentration changes in proportion to the current (Fig. 3C-D, green dotted versus solid curve). Biphasic waveforms (damped oscillations) emerge as the Ca$^{2+}$ kinetics are slowed down (Fig. 3A-B). In this case the Ca$^{2+}$ concentration becomes delayed with respect to the current (Fig. 3C-D, red dotted versus solid curve).

**Fig. 4:**
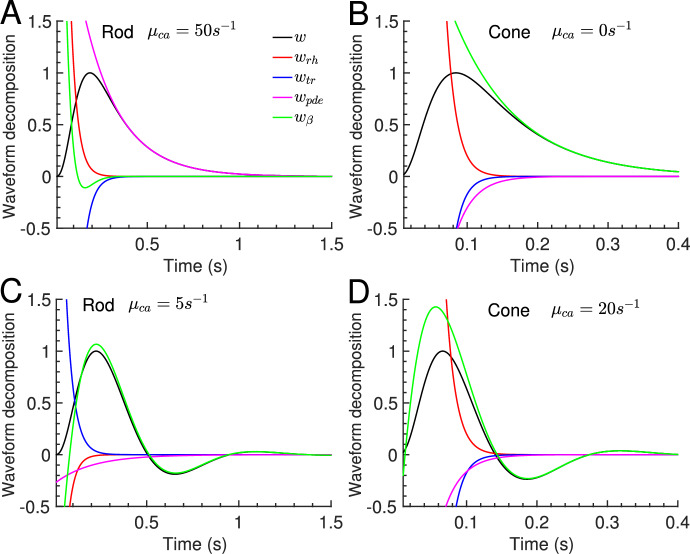
Decomposition of the current waveform of flash responses. The decomposition is obtained with Eq. 17. **A** The recovery in a rod with fast Ca$^{2+}$ dynamics is limited by PDE decay, $w_{pde}\sim e^{-\mu _{pde} t}$. **B** The recovery in a cone with constant Ca$^{2+}$ concentration is limited by $\beta _d$, $w_{\beta }\sim e^{-\beta _d t}$. This is also true for in GCAPs$^{-/-}$ mutant cones where the Ca$^{2+}$ feedback to the cyclase is genetically removed. **C**–**D** The recovery with slow Ca$^{2+}$ kinetics are determined by a damped oscillation

With Eq. 17 we decompose the current waveform into the contributions of the various biophysical processes, $w= w_{rh} + w_{tr} + w_{pde} + w_{\beta }$ (Fig. 4). Such a decomposition cannot be obtained from simulations. The waveform decomposition shows that the recovery in a rod with fast Ca$^{2+}$ kinetics are limited by PDE decay, $w_{pde}\sim e^{-\mu _{pde} t}$ (Fig. 4A). The recovery in a cone with constant Ca$^{2+}$ concentration ($\mu _{ca}=0$) is limited by $\beta _d$, $w_{\beta }\sim e^{-\beta _d t}$ (see the analysis after Eq. 17 for details). This is also the case for the recovery in a GCAPs$^{-/-}$ cone where the cyclase is not Ca$^{2+}$ dependent. Note that the Ca$^{2+}$ current is not constant in a GCAPs$^{-/-}$ mutant, contrary to the case with $\mu _{ca}=0$. However, because the Ca$^{2+}$ current is small, flash responses in GCAPs$^{-/-}$ mutant mice vary only little when the Ca$^{2+}$ dynamics is distorted, contrary to the Wt case (Burns et al. 2002). Except for such special cases, the recovery of a flash response cannot be approximated by a single exponential decay function. For example, because $\mu _{pde}$ and $\beta _d$ have similar values in a mouse rod, the recovery in a GCAPs$^{-/-}$ rod is determined by a sum of two exponentials (see Fig. 4D in Abtout et al. (2021)). As another example, with slow Ca$^{2+}$ dynamics the recovery is determined by a damped oscillation (Fig. 4C and D).

### Peak amplitude of flash responses and photoreceptor sensitivity

**Fig. 5:**
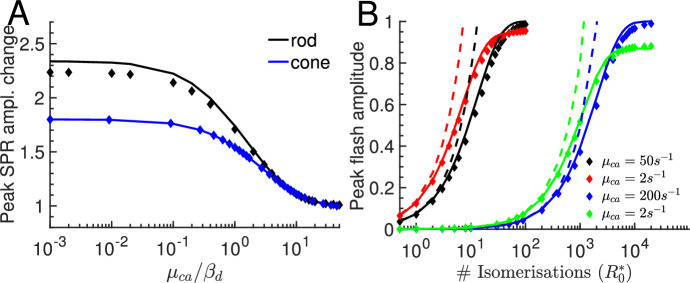
Peak amplitudes of flash responses. **A** Change of the SPR peak amplitude as a function of $\mu _{ca}/\beta _d$ for a rod (black curve and diamonds) and a cone (blue curve and diamonds). The peak amplitude is normalized to the value for fast Ca$^{2+}$ kinetics. Diamonds show values extracted from simulations, solid lines are computed with Eq. 16 and $R_0^*=1$. **B** Peak amplitude as a function of the number of isomerisations $R_0^*$ for slow ($\mu _{ca}=2s^{-1}$) and fast Ca$^{2+}$ kinetics ($\mu _{ca}=50s^{-1}$ for rod and $\mu _{ca}= 200 s^{-1}$ for cone). Black and red colors are for rod, blue and green for cone. Diamonds show values obtained from simulations. Solid lines are computed with ${\hat{i}}_{peak} = 1-e^{-R^*_0 \xi n_{ch} g_{y,peak}}$ for fast Ca$^{2+}$ kinetics, and ${\hat{i}}_{peak} = \frac{2}{f_{ch,ca}+2} \left( 1-e^{-R^*_0 \xi n_{ch} g_{y,peak}}\right)$ for slow kinetics (see text for explanations). The dashed lines show ${\hat{i}}_{peak}$ computed with the linear approximation in Eq. 16 (color figure online)

The Ca$^{2+}$ kinetics affects not only the waveform of a light response, but also the peak amplitude (sensitivity). As the Ca$^{2+}$ kinetics becomes slower, the peak amplitude of the SPR increases by a factor up to 1.8 for a cone, and up to 2.3 for a rod (Fig. 5A). A slower Ca$^{2+}$ kinetics leads to a smaller reduction of the Ca$^{2+}$ concentration at time to peak, a reduced negative cyclase feedback and a larger SPR. A larger SPR also implies a higher light sensitivity. Because the exchanger current is small, the condition with $\mu _{ca}\rightarrow 0$ also characterizes a GCAPSs$^{-/-}$ photoreceptor. Thus, Fig. 5A shows that the difference of the SPR between a WT and GCAPSs$^{-/-}$ photoreceptor depends on the Ca$^{2+}$ kinetics, and the largest difference is attained when the Ca$^{2+}$ kinetics are fast.

In first approximation, the peak current increases linearly with the number of isomerisations $R_0^*$ (Eq. 16). The linear approximation is valid up to ${\hat{i}}_{peak}\sim 0.2-0.3$ (Fig. 5B; dashed lines are the peak current computed with Eq. 16, diamonds are simulation results), which corresponds to few isomerisations for a rod, but few hundreds for a cone (for example, with fast Ca$^{2+}$ kinetics, the peak SPR amplitude in a rod is $\sim 0.07$ and $\sim 4.7\times 10^{-4}$ in a cone). The slight discrepancy in Fig. 5A between the simulation and linear approximation for a rod with slow Ca$^{2+}$ kinetics arises because of the large SPR amplitude ${\hat{i}}_{peak}\sim 0.16$.

To obtain an approximation for the peak current that remains valid up to higher light intensities, we combine the linear results with saturation effects. With $y=R^*_0 \xi g_{y}$ and $z=R^*_0 \xi \nu g_{u}$ we get ${\hat{c}}_{cg} = e^{-y}=e^{-R^*_0 \xi g_{y}}$, ${\hat{c}}_{ca} = e^{-z}=e^{-R^*_0 \xi \nu g_u}$, ${\hat{p}}_{ch} \approx e^{-R^*_0 n_{ch}\xi g_{y}}$, and ${\hat{p}}_{ex} \approx e^{-R^*_0 n_{ex}\xi \nu g_{u}} \approx e^{-R^*_0 n_{ch}\xi g_{u}}$, where we used ${\hat{K}}_{ch}\gg 1$ and ${\hat{K}}_{ex}\gg 1$. With Eq. 8 we get ${\hat{i}} = 1- \frac{2}{f_{ch,ca}+2}{\hat{p}}_{ch} - \frac{f_{ch,ca}}{f_{ch,ca}+2} {\hat{p}}_{ex} \approx 1- \frac{2}{f_{ch,ca}+2} e^{-R^*_0 \xi n_{ch} g_{y}} - \frac{f_{ch,ca}}{f_{ch,ca}+2} e^{-R^*_0 \xi n_{ch} g_{u}}$. With fast Ca$^{2+}$ kinetics we have $g_y\approx g_u$ (the solution of Eq. 11 for $\mu _{ca}\rightarrow \infty$ is $y=u$) and ${\hat{i}}_{peak} \approx 1-e^{-R^*_0 \xi n_{ch} g_{y,peak}}$. With slow kinetics the Ca$^{2+}$ concentration and the exchanger current at peak time are only little affected ($g_u(t_{peak})\approx 0$) and ${\hat{i}}_{peak} \approx \frac{2}{f_{ch,ca}+2} \left( 1-e^{-R^*_0 \xi n_{ch} g_{y,peak}}\right)$. These expressions for ${\hat{i}}_{peak}$ for fast and slow Ca$^{2+}$ kinetics are now in good agreement with simulation results for rods and cones up to saturating flashes (Fig. 5B, diamonds versus solid lines). The numerical values for $g_{y,peak}$ used in Fig. 5B are computed with Eq. 15 and parameters from Table 1: for $\mu _{ca}=2s^{-1}$ we have $g_{y,peak}\approx 0.32$ for a rod and $g_{y,peak}\approx 0.52$ for a cone; for a rod with $\mu _{ca}=50s^{-1}$ we have $g_{y,peak}\approx 0.17$, and for a cone with $\mu _{ca}=200s^{-1}$ we have $g_{y,peak}\approx 0.28$. Since $g_{y,peak}$ is not much different between rods and cones, the higher sensitivity of a rod to a pigment activation is due to the larger transduction gain $\xi$. With parameters from Table 1 we find $\frac{\xi _{rod}}{\xi _{cone}}\approx 250$, which corresponds to the sensitivity gap between rods and cones in Fig. 5B. The sensitivity of a photoreceptor increases with slower Ca$^{2+}$ kinetics because of reduced negative Ca$^{2+}$ feedback at time to peak, in agreement with Fig. 5A. With slow Ca$^{2+}$ kinetics the exchanger current does not contribute at peak time, and flash responses saturate at $\frac{2}{f_{ch,ca}+2}$ (also the saturating flash responses in Fig. 1B,D that do not reach the maximal value of one). This effect is more pronounced in a cone due to a larger exchanger current and higher value $f_{ch,ca}$. For intermediate Ca$^{2+}$ kinetics we do not have and analytic approximation; in this case one has to estimate the peak time $t_{peak}$, and then compute ${\hat{i}}_{peak} \approx 1- \frac{2}{f_{ch,ca}+2} e^{-R^*_0 \xi n_{ch} g_y(t_{peak})} - \frac{f_{ch,ca}}{f_{ch,ca}+2} e^{-R^*_0 \xi n_{ch} g_u(t_{peak})}$.

### Phase space analysis and the emergence of damped oscillations

In section 2.2.2 we showed that the photoresponse contains a damped oscillation if the ratio $\rho = \frac{\mu _{ca}}{\beta _d}$ is within the range $\rho _{1}< \rho < \rho _{2}$, where $\rho _1 = \frac{1+{\hat{K}}_{ex}^{n_{ex}}}{n_{ex}{\hat{K}}_{ex}^{n_{ex}}} \left( 1+2\nu {\hat{\alpha }}'_0 \left( 1- \sqrt{1+ \frac{1}{\nu {\hat{\alpha }}'_0 } } \right) \right)$ and $\rho _2 = \frac{1+{\hat{K}}_{ex}^{n_{ex}}}{n_{ex}{\hat{K}}_{ex}^{n_{ex}}} \left( 1+2\nu {\hat{\alpha }}'_0 \left( 1+ \sqrt{1+ \frac{1}{\nu {\hat{\alpha }}'_0 }} \right) \right)$. The values $\rho _1$ and $\rho _2$ depend on CNG and exchanger properties and the cyclase feedback ${\hat{\alpha }}_0'$. With values from Table 1 we find $\nu \approx 2.9$, ${\hat{\alpha }}'_0\approx 1.05$, $\rho _1 \approx 0.08$ and $\rho _2 \approx 16.9$.

**Fig. 6:**
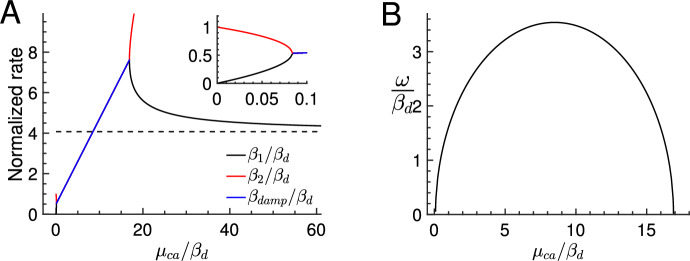
Phase space analysis for Ca$^{2+}$ oscillations. **A** The normalized exponential decay rates $\beta _1= \beta _d \lambda _1$ (black line) and $\beta _2= \beta _d \lambda _2$ (red line) for the current component ${\hat{i}}_\beta$ in Eq. 17 are shown as a function of $\frac{\mu _{ca}}{\beta _d}$ (the inset magnifies the region for small $\mu _{ca}$). The dashed black line shows the asymptotic value $\frac{\beta _1}{\beta _d} = 1 +\nu {\hat{\alpha }}'_0$ for $\mu _{ca}\rightarrow \infty$. Damped oscillations occur for $0.08< \frac{\mu _{ca}}{\beta _d} < 16.9$ (see Sect.  3.3). In this range the eigenvalues $\lambda _1$ and $\lambda _2$ are complex conjugate, and the damping rate is $\beta _{damp}\approx \beta _d \frac{1 +\frac{\mu _{ca}}{\beta _d}}{2}$ (blue line). **B** The normalized frequency $\omega$ of the damped oscillation as a function of $\frac{\mu _{ca}}{\beta _d}$. The maximal frequency $\omega _{max} \approx \beta _d \frac{16.9}{4}$ is attained for $\frac{\mu _{ca}}{\beta _d} \approx \frac{16.9 }{2}$. All values have been computed with parameters from Table 1

For $\rho >\rho _2$ or $\rho <\rho _1$, the current ${\hat{i}}_{\beta }= R^*_0 c_{\beta _1}e^{-\beta _1t} + R^*_0 c_{\beta _2} e^{-\beta _2t}$ to ${\hat{i}}$ in Eq. 17 is a sum of two exponentials with decay rates $\beta _1=\beta _d \lambda _1$ and $\beta _2=\beta _d \lambda _1$ (Fig. 6A black and red curves). For $\mu _{ca}\rightarrow 0$ we have the asymptotic relation ${\hat{i}}_{\beta }\sim e^{-\beta _2 t} = e^{-\beta _d t}$, whereas for fast Ca$^{2+}$ kinetics ($\mu _{ca}\rightarrow \infty$) we find ${\hat{i}}_{\beta }\sim e^{-\beta _1 t} = e^{-\beta _d(1 +\nu {\hat{\alpha }}'_0) t}$, with $1 +\nu {\hat{\alpha }}'_0 \approx 4$.

If $\rho$ is between $\rho _1=0.08$ and $\rho _2=16.9$, the rates $\beta _1$ and $\beta _2$ are complex conjugate and ${\hat{i}}_{\beta }$ is a damped oscillation with damping rate $\beta _{damp}\approx \beta _d \frac{1 + \rho }{2}$ (Fig. 6A blue line) and oscillation rate $\omega \approx \beta _d \frac{\sqrt{(\rho -\rho _1)(\rho _2-\rho )} }{2}$ (Fig. 6B) (we assumed $\frac{n_{ex}{\hat{K}}_{ex}^{n_{ex}}}{1+{\hat{K}}_{ex}^{n_{ex}}}\approx 1$ such that $r\approx \rho$)). The maximal frequency $\omega _{max} \approx \beta _d \frac{\rho _2}{4}$ is attained for $\rho \approx \frac{\rho _2 }{2}$ (Fig. 6B).

Although the photoresponse contains a damped oscillation over a wide range of $\mu _{ca}$ values, the oscillation can be observed during the recovery phase only if the damping rate $\beta _{damp}$ is not too large. For example, with $\rho _2= 16.9$ we compute that oscillations are present for $\mu _{ca} \lesssim \rho _2 \beta _d \approx 69\,s^{-1}$ in a rod and $\mu _{ca} \lesssim 186\,s^{-1}$ in a cone. However, the rod waveform clearly exhibits an oscillation only for $\mu _{ca} \lesssim 10 s^{-1}$ (Fig. 3A), and the cone waveforms for $\mu _{ca} \lesssim 50 s^{-1}$ (Fig. 3B). It is difficult to give an estimate for the value of $\mu _{ca}$ that characterizes when oscillations start to affect the recovery phase, since this depends on the damping rate, the initial oscillation amplitude, and other rate constants that affect the recovery.

**Fig. 7:**
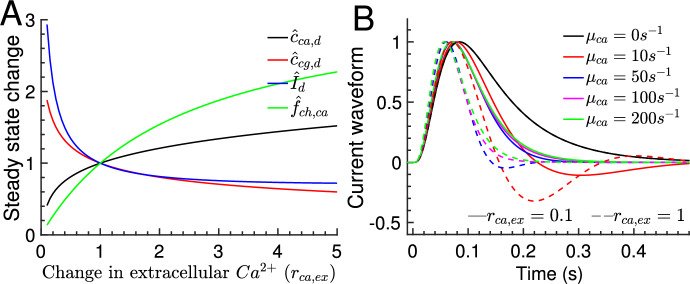
Effect of changing the extracellular Ca$^{2+}$ concentration for a cone. **A** Changes of the dark steady state concentrations of Ca$^{2+}$ (black curve) and cGMP (red curve), the dark current (blue curve) and the fraction of the CNG current that is carried by Ca$^{2+}$ (green curve) when the extracellular Ca$^{2+}$ concentration is altered by a factor of $r_{ca,ex}$. ${\hat{c}}_{ca,d}$ has been computed numerically by solving the steady state of Eq. 22; ${\hat{c}}_{cg,d}={\hat{\alpha }} ({\hat{c}}_{ca,d})$; ${\hat{I}}_d$ is computed with Eq. 23; ${\hat{f}}_{ch,ca}=\frac{f'_{ch,ca}}{f_{ch,ca}}$ is computed with Eq. 20. **B** Comparison of current waveforms computed with Eq. 16 for $r_{ca,ex}=0.1$ (solid lines) and $r_{ca,ex}=1$ (dashed lines) and for various values of $\mu _{ca}$ as indicated in the legend (the dashed curves are as in Fig. 3B; dashed and solid curve overlay for $\mu _{ca}=0s^{-1}$) (color figure online)

### Effect of changing the extracellular Ca$^{2+}$ concentration

We investigated the effect of changing the extracellular Ca$^{2+}$ concentration by a factor of $r_{ca,ex}$ for a cone. Similar conclusions are obtained for a rod (not shown). In the first place, changing the extracellular Ca$^{2+}$ concentration alters the fraction of the CNG current that is carried by Ca$^{2+}$ (Fig. 7A, green curve), which changes the dark steady state concentrations of Ca$^{2+}$ and cGMP (Fig. 7A, black and red curve), and the dark current (Fig. 7A, blue curve). For example, with parameters from Table 1 we find that when the extracellular Ca$^{2+}$ concentration is reduced by tenfold ($r_{ca,ex}=0.1$), the fraction of the CNG current carried by Ca$^{2+}$ and the dark steady state concentration of Ca$^{2+}$ are reduced by a factor of around 7.3 and 2.5, respectively. The lower Ca$^{2+}$ concentration activates the cyclase, which results in a cGMP concentration that is elevated by a factor of around 1.9, and an dark current that is increased by a factor of around 2.9.

To estimate how the Ca$^{2+}$ kinetics governed by $\mu _{ca}$ (Eq. 6) is affected by extracellular Ca$^{2+}$, we note that the ratio $\frac{I_{ex}}{c_{ca}}$ is independent of the Ca$^{2+}$ concentration for the physiological values $n_{ex}=1$ and ${\hat{K}}_{ex} \gg 1$. Thus, the impact on the Ca$^{2+}$ kinetics depends on the change of the buffering capacity $B_{ca}$. If the low affinity buffer recoverin is prevalent, $B_{ca}$ is not much altered by reducing extracellular Ca$^{2+}$. In contrast, with high affinity buffers the value of $B_{ca}$ increases. For example, for a buffer with dissociation constant $K_{b_2}= 0.14 \mu M$, a reduction of the dark-adapted Ca$^{2+}$ concentration from $0.3 \mu M$ to $0.15\mu M$ due increases the buffering capacity $B_{b_2}$ by a factor of 2.3. Assuming that low and high affinity buffers contributed equally with $c_{ca}=0.3 \mu M$, it follows that $B_{ca}$ increases by a factor of $(1+2.3)/2\sim 1.65$ if the dark-adapted Ca$^{2+}$ concentration drops to $c_{ca}=0.15 \mu M$, which reduces the rate $\mu _{ca}$ by a factor of around 0.6.

Changing the extracellular Ca$^{2+}$ concentration strongly affects dimensional current amplitude (measured in units of *pA*) because the latter is proportional to the dark current. This scaling effect can be removed by normalizing responses with the corresponding dark current. The normalized flash response in Eq. 16 changes only moderately, which is mostly to the modified cyclase feedback ${\hat{\alpha }}_0'$. For example, with parameters from Table  1 we compute ${\hat{\alpha }}_0'=1.05$ for $r_{ca,ex}=1$, and ${\hat{\alpha }}_0'=0.33$ for $r_{ca,ex}=0.1$. The reduced cyclase feedback ${\hat{\alpha }}_0'$ increases the normalized SPR amplitude by a factor between 1 and 1.5 (not shown) depending on the Ca$^{2+}$ kinetics (the strongest effect is obtain with fast Ca$^{2+}$ kinetics). In contrast, the dimensional current is additionally scaled by a factor around 2.9 due to the change in the dark current.

The reduced cyclase feedback ${\hat{\alpha }}_0'=0.33$ with $r_{ca,ex}=0.1$ also affects the current waveform, leading to a longer time to peak, reduced oscillations and a slower recovery (Fig. 7B, solid versus dashed curves). The waveforms in Fig. 7B for $r_{ca,ex}=1$ and $r_{ca,ex}=0.1$ have been computed with the values of $\mu _{ca}$ specified in the figure legend. Thus, if $B_{ca}$ and $\mu _{ca}$ are also changed by reducing extracellular Ca$^{2+}$, one has to compare the waveforms with the corresponding values of $\mu _{ca}$.

### Responses to steps of light

**Fig. 8:**
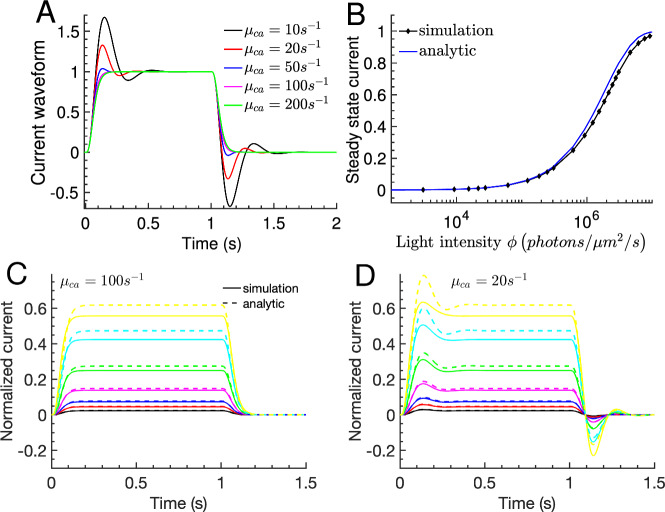
Responses to steps of light for a cone. **A** The dim-light current waveform from Eq. 24 for steps with duration $\Delta t =1s$ and for various values of $\mu _{ca}$ as indicated in the legend. **B** The steady state current from Eq. 25 (blue curve) as a function of the light intensity $\phi$ is compared to results obtained by numerically computing the steady state with Eq. 8 (black line with markers). **C** and **D** Simulations of step responses (solid lines) are compared to the corresponding analytic result computed with Eq. 26 (dashed lines) for fast **C** and slow **D** Ca$^{2+}$ kinetics. The light stimulations in **C** and **D** are identical (color figure online)

Finally, we considered the photoreceptor response to steps of light with intensity $\phi$ and duration $\Delta t$ for a cone. Similar results are obtained for a rod (not shown). For sufficiently long step duration $\Delta t$, the initial rising phase of the response is followed by an intermediate steady state phase (plateau phase), and a recovery phase after the light is switched off. Because the plateau current depends on $\phi$ but is independent of $\mu _{ca}$, we define the waveform by normalizing a step response with its plateau current. With the steady state solution $g_{y,ss}=g_{u,ss}=\frac{1}{\beta _d(1+ \nu {\hat{\alpha }}'_0) \Delta t}$, the dim-light current waveform of a step response is given by

24 $$\begin{aligned} w=\beta _d(1+ \nu {\hat{\alpha }}'_0) \Delta t \left( \frac{2}{f_{ch,ca}+2} g_y +\frac{f_{ch,ca}}{f_{ch,ca}+2} g_u \right) . \end{aligned}$$

The dynamics during initial and recovery phase depend on $\mu _{ca}$, and oscillations emerge as the Ca$^{2+}$ dynamics is slowed down (Fig. 8A). Whereas the flash waveform differs between initial and recovery phase (Fig. 3A-B), there is a symmetry between these phases for step responses (Fig. 8A). Indeed, with Eq. 11 we find ${\hat{i}}(\Delta t + t) = {\hat{i}}_{ss} - {\hat{i}}(t)$, where ${\hat{i}}_{ss}$ is the intermediate steady state current.

With the steady state relation ${\hat{i}}= 1 -{\hat{p}}_{ch}$ and the first order result $y=R^*_0 \xi g_{y}$ we get for cGMP ${\hat{c}}_{cg,ss}=e^{-R^*_0 \xi g_{y,ss}}$, and for the current (${\hat{K}}_{ch}\gg 1$)

25 $$\begin{aligned} {\hat{i}}_{ss} = 1-e^{-\frac{ n_{ch} \kappa \xi \phi }{\beta _d(1+ \nu {\hat{\alpha }}'_0)} }. \end{aligned}$$

We find that Eq. 25 well approximates the results obtained by numerically solving the steady state of Eq. 8 up to saturating light intensities (Fig. 8B, blue versus black curve). For example, with parameters from Table 1 we compute $\left( \frac{\kappa \xi n_{ch}}{\beta _d(1+ \nu \hat{\alpha }'_0)}\right) ^{-1} \approx 1.9\times 10^{6} \frac{photon}{\mu m^2\,s}$ for a cone, and $132 \frac{photon}{\mu m^2\,s}$ for a rod. Equation 25 shows that the steady state current in a GCAPs$^{-/-}$ photoreceptor with ${\hat{\alpha }}'_0=0$ is by a factor of $1+ {\tilde{n}}_{ch} {\hat{\alpha }}'_0\approx 4$ larger compared to WT. This difference is much larger than a value around 1.8 found for the difference in the SPR amplitude (Fig. 5A, blue curve).

Finally, by combining Eq. 24 with Eq. 25 we obtain for a step response

26 $$\begin{aligned} {\hat{i}} = \left( 1-e^{-\frac{n_{ch} \kappa \xi \phi }{\beta _d(1+ \nu {\hat{\alpha }}'_0)} }\right) \beta _d(1+ \nu {\hat{\alpha }}'_0) \Delta t \left( \frac{2}{f_{ch,ca}+2} g_y +\frac{f_{ch,ca}}{f_{ch,ca}+2} g_u \right) . \end{aligned}$$

Equation 26 is in good agreement with simulation results up to almost half saturating responses for fast Ca$^{2+}$ kinetics with $\mu _{ca}=100s^{-1}$ (Fig. 8C, dashed versus solid curves), as well as slow kinetics with $\mu _{ca}=20s^{-1}$ (Fig. 8D, dashed versus solid lines).

## Discussion

The signal transduction pathway in the outer segment of rod and cone photoreceptors consists of a series of biophysical processes that transform light into an electrical current. Many of these processes are modulated by Ca$^{2+}$ feedback, which affects response dynamics and photoreceptor sensitivity. To obtain conceptual and quantitative insight that goes beyond numerical simulations, we studied the light response with a parsimonious model that allows for a comprehensive mathematical analysis. The model includes the principal transduction features that are known to be relevant for the light response under dark-adapted conditions. It incorporates fast buffering reactions to alter the Ca$^{2+}$ kinetics, and negative Ca$^{2+}$ feedback onto the synthesis of cyclic GMP, which is the most important feedback for dim light (Burns et al. 2002; Sakurai et al. 2011; Koutalos et al. 1995). To obtain analytic results, we performed a linear response analysis for dim light conditions where the current change is small compared to the dark current. The current response is determined by $\phi \kappa \xi$ (see Eq. 5), and since collecting area $\kappa$ and transduction gain $\xi$ are much smaller in cones compared to rods (Table 1), the linear response analysis remains valid up to much higher light intensities $\phi$ in cones compared to rods. Thus, the definition of dim light depends on the photoreceptor type. For example, dim light for a cone can induce already saturating responses in a rod (Fig. 5B). We combined the analytic results with numerical simulations to obtain quantitative insight about how the various biophysical processes and the Ca$^{2+}$ kinetics determine waveform and amplitude of flash and step responses. We further investigated how the light response is affected by changing the extracellular Ca$^{2+}$ concentration.

The Ca$^{2+}$ kinetics with fast buffering is governed by the effective rate $\mu _{ca}= \frac{|I_{ex}|}{(1+B_{ca})F_A V_{os}c_{ca}}$ (Eq. 6). $V_{os}$ is the outer segment volume, $c_{ca}$ is the steady state concentration of free Ca$^{2+}$, $I_{ex}$ is the steady state exchanger current, $F_A$ is the Faraday constant, and $B_{ca}$ is the cumulated buffering capacity that depends on the various buffer species. Because $I_{ex}$ is proportional to $c_{ca}$ for $K_{ex} \gg c_{ca}$ (see Table 1), it follows that $\frac{I_{ex}}{c_{ca}}$ only depends on intrinsic exchanger properties and their density in the membrane. In contrast, the buffering capacity of $B_{ca}$ depends on the Ca$^+$ affinities of the individual buffers and therefore the steady state concentration of Ca$^+$, which further depends on the background light intensity. The most abundant Ca$^{2+}$ buffer in the outer segment is recoverin (Pugh and Lamb 2000; Kawamura and Tachibanaki 2021) with a dissociation constant $K_{ca,rec}\sim 3\mu M$ (Chen et al. 1995) that is much larger than the dark-adapted steady state concentration of Ca$^+$ around $0.3\mu M$ (Table 1). With such conditions we conclude that $B_{ca}$ and the Ca$^+$ kinetics are not much affected by light as the Ca$^+$ concentration further decreases. But $B_{ca}$ and the Ca$^{2+}$ kinetics can be altered by adding exogenous buffers (Torre et al. 1986; Korenbrot and Miller 1989; Rieke and Baylor 1998; Field and Rieke 2002; Burns et al. 2002; Matthews 1991; Burns et al. 2002; Makino et al. 2004).

We define the waveform by normalizing a flash response with its peak amplitude. The waveform therefore characterizes the dynamics of a response (Fig. 2). With fast Ca$^{2+}$ kinetics, the Ca$^{2+}$ concentration changes in proportion to the current and the waveforms are monophasic (Fig. 3). As the Ca$^{2+}$ kinetics are slowed down due to more buffering, the Ca$^{2+}$ concentration becomes delayed with respect to the current and biphasic waveforms emerge (Fig. 3). The biphasic shape is due to a damped oscillation generated by the negative feedback interaction between Ca$^{2+}$ and cGMP synthesis. Since we only consider fast buffering, this shows that biphasic responses are not necessary an indication of slow buffering reactions. The analysis reveals that the presence of damped oscillations depends on the ratio $\mu _{ca}/\beta _d$, where $\beta _d$ is the dark turnover rate of cGMP (Fig. 6A). This is consistent with results from a parameter sensitivity analysis in cones, which identified $\beta _d$ and exchanger properties (which affects $\mu _{ca}$) as most important for the presence of biphasic responses (Klaus et al. 2021). With parameters from Table 1 we find that a damped oscillation is present if $\mu _{ca}/\beta _d$ is below an upper threshold of around 17, and above a lower threshold of around 0.08. These values depend on the strength of Ca$^{2+}$ feedback to the cyclase $\hat{\alpha }'_0$, and the channel cooperativity $n_{ch}$ (see Sect. 3.3). Oscillations disappear for $\mu _{ca}\rightarrow 0$ (Fig. 3A) because in this limit the Ca$^{2+}$ concentration remains constant and no negative feedback to the cyclase occurs. Oscillations are also absent in GCAPs$^{-/-}$ mutant mice where the cyclase is not Ca$^{2+}$-dependent (Mendez et al. 2001; Burns et al. 2002). The oscillation frequency $\omega$ and the damping rate $\beta _{damp}$ depend on $\mu _{ca}$ (Fig. 6A,B). Whereas the oscillation frequency first increases and then decreases as a function of $\mu _{ca}$ (Fig. 6B), the damping rate steadily increases with faster Ca$^{2+}$ kinetics (Fig. 6A). Although oscillations are present over a wide range of $\mu _{ca}$ values, they affect the recovery phase only if the damping and therefore the Ca$^{2+}$ kinetics are not too fast. Because oscillation properties depend on the ratio $\mu _{ca}/\beta _d$, the same Ca$^{2+}$ kinetics does not necessarily lead to similar waveforms because $\beta _d$ differs between rods, cones, and animal species (Pugh and Lamb 2000). For example, $\beta _d$ is around 3-fold larger in a mouse cone compared to mouse rod (Reingruber et al. 2020). The difference in $\beta _d$ might be a reason why oscillations have been rarely observed in amphibian compared to primate rods (Tamura et al. 1991), or why oscillations have been more frequently observed in Wt cones compared to Wt rods (Schneeweis and Schnapf 1999; Schnapf et al. 1990; Baylor et al. 1987).

With our analytic results we find that the waveform can be decomposed into a sum of exponentials that reflect the underlying biophysical processes (Eq. 17 and Fig. 4). Such a decomposition cannot be performed with simulations only. We used the decomposition to study which processes limit the response recovery. For example, the recovery of flash responses in a WT mouse rod with fast Ca$^{2+}$ kinetics are limited by PDE decay (Fig. 4A), in agreement with experimental findings (Krispel et al. 2006; Tsang et al. 2006; Chen et al. 2010). In a GCAPs$^{-/-}$ cone the recovery is limited by $\beta _d$ (Fig. 4B). For these special cases it is possible to extract parameters by fitting the response recovery with a single exponential decay function. However, in general, the recovery of flash responses is not governed by a single exponential. For example, for a GCAPs$^{-/-}$ rod the recovery depends on the PDE decay rate $\mu _{pde}$ and the dark turnover rate $\beta _d$ (see Fig. 4D in Abtout et al. (2021)). And with sufficiently slow $Ca^{2+}$ dynamics the recovery is is not an exponential decay but a damped oscillation (Fig. 4C-D).

The Ca$^{2+}$ kinetics not only affect the waveform, but also the photoreceptor sensitivity characterised by the peak current amplitude evoked by dim flashes. The amplitude of the single-photon response (SPR) increases as the Ca$^{2+}$ kinetics are slowed down because negative Ca$^{2+}$ feedback is delayed and less strong at peak time (Fig. 5A). This finding explains the larger photoreceptor sensitivity observed with exogenous buffering (Matthews 1991). The sensitivity change between Wt and GCAPs$^{-/-}$ photoreceptors is maximal with fast the Ca$^{2+}$ kinetics (Fig. 5A). With parameters from Table 1 we compute a maximal change around 2.3 for a rod, and around 1.8 for a cone (Fig. 5A). These values can change depending on the model for the cyclase. The experimentally observed sensitivity change between GCAPs$^{-/-}$ and Wt mouse rods is found to be around 2-3 (Reingruber et al. 2020; Burns et al. 2002; Mendez et al. 2001), which suggests that the physiological Ca$^{2+}$ kinetics are fast and the Ca$^{2+}$ concentration changes in proportion to the current, in agreement with (Li et al. 2020; Matthews and Fain 2003).

The peak flash amplitude ${\hat{i}}_{peak}$ depends on the number of isomerisations $R_0^*=\kappa \phi \Delta t$ produced by the flash. In the past, the empirical function ${\hat{i}}_{peak}=1-e^{-a \phi \Delta t}$ has been often used to model the flash amplitude as a function of the flash strength $\phi \Delta t$, and to estimate the flash sensitivity *a* by fitting the data (Morshedian et al. 2022, 2017; Astakhova et al. 2015; Korenbrot 2012; Chen et al. 2010; Tranchina et al. 1991; Schnapf et al. 1990; Nakatani and Yau 1988; Baylor et al. 1984; Schnapf et al. 1987; Baylor et al. 1987). A possible explanation for this empirical a function has been given based on a spatial model where the probability of channel closure depends on the statistical superposition of isomerisations along the outer segment (the total occlusion model) (Lamb et al. 1981), or based on a non-spatial model with pigment bleaching (Hodgkin and Obryan 1977). We showed ${\hat{i}}_{peak}=1-e^{-a \phi \Delta t}$ is a valid approximation for the peak amplitude for fast Ca$^{2+}$ kinetics (Fig. 5B), and we computed the sensitivity as function the biophysical parameters $a=\kappa \xi n_{ch} g_{y,peak}$: $\kappa$ is the collecting area, $\xi$ is the transduction gain, $n_{ch}$ is the CNG channel cooperativity, and $g_{y,peak}$ depends on the dynamics and can be computed from Eq. 15. With parameters from Table 1 we estimate $a=0.02$ for a mouse rod (*a* has units $\frac{\mu m^2}{photons}$), which is consistent with 0.014 (Morshedian et al. 2017) and 0.026 (Chen et al. 2010) obtained by fitting experimental data. For a mouse cone we compute a much lower sensitivity $a=0.65\times 10^{-5}$, compatible with (Ingram et al. 2019). Since the values of $g_{y,peak}$ are not much different between rods and cones, the large sensitivity gap between rods and cones is due to collecting area $\kappa$ and the gain $\xi$. Since collecting area and gain vary among species, this leads to variations in the sensitivity *a*. For example, a sensitivity of 0.03 has been estimated for monkey rods (Baylor et al. 1984), $2.77 \times 10^{-4}$ and $3 \times 10^{-5}$ for monkey cones (Baylor et al. 1987), $\sim 10^{-3}$ for human cones (Schnapf et al. 1987), and $7.3 \times 10^{-3}$ for a bass cone (Korenbrot 2012).

We explored how changing the extracellular Ca$^{2+}$ concentration affects the light response in a cone (similar conclusions are obtained for a rod (not shown)). Modifying the extracellular Ca$^{2+}$ concentration alters the Ca$^{2+}$ influx and the fraction of the CNG current that is carried by Ca$^{2+}$ (Eq. 20 and Fig. 7A, green line). This changes the steady state concentrations of Ca$^{2+}$ and cGMP, and the dark current (Fig. 7A). For example, a tenfold reduction in the extracellular Ca$^{2+}$ concentration reduces the Ca$^{2+}$ influx by a factor of around 7.3, and the dark-adapted Ca$^{2+}$ concentration in the OS by a factor of around 2.5. This activates the cyclase and increases the dark-adapted cGMP concentration by a factor of around 1.9, and the dark current by a factor of around 2.9. But the reduction in extracellular Ca$^{2+}$ also slows down the dynamics, increases the photoreceptor sensitivity, and reduces the amplitude of oscillations (Fig. 7B). Similar effects as described here have been observed experimentally for primate cones when the extracellular Ca$^{2+}$ concentration has been reduced around tenfold (see Fig. 2A in Cao et al. (2014)).

For a cone we also studied how the Ca$^{2+}$ kinetics affects the responses to steps of light (Fig. 8) (similar conclusions apply for a rod, not shown). The Ca$^{2+}$ kinetics affects step responses only during initial and recovery phase, whereas the intermediate plateau phase depends on steady state properties that are independent of $\mu _{ca}$. For dim light, the recovery phase is a mirror image of the initial phase (Fig. 8A). Hence, whereas initial and recovery phase of dim-flash responses are different and provide complementary information (Fig. 2), this is not the case for step responses. We derived an exponential approximation for steady state current ${\hat{i}}_{ss}$ (which is the intermediate plateau current) as function of the background light intensity $\phi$ (Eq. 25 and Fig. 8B). With Eq. 25 we estimate that the light intensity $\phi _{1/2}$ for which ${\hat{i}}_{ss}=0.5$ is $\phi _{1/2} = \ln 2 \frac{\beta _d(1+ \nu {\hat{\alpha }}'_0)}{\kappa \xi n_{ch}}\sim 1.3\times 10^6 \frac{photon}{\mu m^2\,s}$, where we used parameters from Table. 1. With the collecting area $\kappa =0.013\mu m^2/photons$ we find $\kappa \phi _{1/2} \sim 1.7 \times 10^4 \frac{R^*}{s}$ visual pigment activations per second. Since the value is around tenfold smaller than $2.5 \times 10^5 \frac{R^*}{s}$ extracted from steady state recordings (Ingram et al. 2019), this indicates that our model saturates too quickly with increasing background light intensity. The most plausible explanation is that this model lacks adaptation processes that affect the steady state at higher background light intensities, for example accelerated photopigment and PDE deactivation, and photopigment bleaching (Fain et al. 2001; Fain 2011). Whereas these processes are important for steady state computations in bright light, they can be neglected for dark-adapted flash responses.

Although in this work we have ignored slow buffering reactions, conceptually it is straightforward to include such reactions in future work, and to generalize the linear response analysis. For example, with a single slow buffering reaction, Eq. 11 will have to be replaced by 3-dimensional system of equations. Although the phase space now becomes more complex, we do not expect to find solutions that are qualitatively very different from what we have described here, consistent with simulations results in presence of slow buffer (Forti et al. 1989; Tamura et al. 1991).

We considered a non-spatial model for the outer segment to be able to derive analytic results. Such models are frequently used to study the photoresponse (Reingruber et al. 2020; Beelen et al. 2021; Astakhova et al. 2015; Invergo et al. 2014; Korenbrot 2012; Chen et al. 2010; Hamer et al. 2005; Moriondo and Rispoli 2003; Nikonov et al. 2000, 1998; Sneyd and Tranchina 1989; Forti et al. 1989). Whereas 3D spatial models (Klaus et al. 2021; Bisegna et al. 2008), or effective 1D longitudinal models derived with the assumption of rapid radial equilibration (Reingruber et al. 2013; Lamb and Kraft 2016; Gross et al. 2012a; Pugh and Lamb 1992), provide a more accurate description of reality, the trade-off is that they are usually studied only with simulations. Nevertheless, we performed simulations with our spatial model from (Reingruber et al. 2013) to check that results are not significantly altered with a spatially extended OS (not shown). Recently it has been found that the large outer segment of a frog rod is a spatially inhomogeneous compartment (Li et al. 2020; Mazzolini et al. 2015). It remains unclear whether this is also true for the much smaller outer segment of mouse or primate rods and cones. We leave it for future work to investigate how a possible spatial inhomogeneity affects the photoresponse.

In this work we used a deterministic mean-field model to derive analytic results that provided functional insight about how the calcium kinetics affects waveform and amplitude of the light response. We neglected noise generated by the PDE activation cascade, for example due to spontaneous PDE activations or low numbers of activated proteins. Since the equations for PDE activation are linear, our mean-field results faithfully characterize averaged responses of a model with stochastic PDE activations. The analytic results are important to understand the simulations obtained with much more complex stochastic models. In future work, stochastic modelling will show how the calcium kinetics affects the background noise and the variability of individual (not averaged) light responses (see, for example, (Hamer et al. 2003; Caruso et al. 2010; Reingruber et al. 2013, 2020)).

We validated our analytic results for different Ca$^{2+}$ kinetics using the physiological parameters from Table 1. We did not perform a more general analysis to explore rates of error between our linearized and nonlinearized model (Epstein 2018; Strogatz 2018; Ruelle 2009), for example, due to variability in the parameter values found in the literature. We leave it for future work to investigate the implications of a larger parameter space, see for example the sensitivity analysis in (Klaus et al. 2021).

Although we focused on dark-adapted photoreceptors, the analysis outlined in this work can be generalized to study flash responses in presence of a background light. To do so, one has to first numerically compute the steady state corresponding to a background light (this can be done with a more complex model that includes adaption processes), and then use the steady state values as input for a linear response analysis.
